# Oncogene *EVI1* drives acute myeloid leukemia via a targetable interaction with CTBP2

**DOI:** 10.1126/sciadv.adk9076

**Published:** 2024-05-15

**Authors:** Dorien Pastoors, Marije Havermans, Roger Mulet-Lazaro, Duncan Brian, Willy Noort, Julius Grasel, Remco Hoogenboezem, Leonie Smeenk, Jeroen A. A. Demmers, Michael D. Milsom, Tariq Enver, Richard W. J. Groen, Eric Bindels, Ruud Delwel

**Affiliations:** ^1^ Department of Hematology, Erasmus MC Cancer Institute, Rotterdam, Netherlands.; ^2^ Oncode Institute, Utrecht, Netherlands.; ^3^ Stem Cell Group, UCL Cancer Institute, University College London, London, UK.; ^4^ Department of Hematology, Amsterdam UMC location Vrije Universiteit Amsterdam, Amsterdam, Netherlands.; ^5^ Cancer Center Amsterdam, Cancer biology and immunology, Amsterdam, Netherlands.; ^6^ Heidelberg Institute for Stem Cell Technology and Experimental Medicine (HI-STEM gGmbH), 69120 Heidelberg, Germany.; ^7^ Division of Experimental Hematology, German Cancer Research Center, DKFZ69120 Heidelberg, Germany.; ^8^ Proteomics Center, Erasmus MC, Rotterdam, Netherlands.

## Abstract

Acute myeloid leukemia (AML) driven by the activation of *EVI1* due to chromosome 3q26/*MECOM* rearrangements is incurable. Because transcription factors such as EVI1 are notoriously hard to target, insight into the mechanism by which EVI1 drives myeloid transformation could provide alternative avenues for therapy. Applying protein folding predictions combined with proteomics technologies, we demonstrate that interaction of EVI1 with CTBP1 and CTBP2 via a single PLDLS motif is indispensable for leukemic transformation. A 4× PLDLS repeat construct outcompetes binding of EVI1 to CTBP1 and CTBP2 and inhibits proliferation of 3q26/*MECOM* rearranged AML in vitro and in xenotransplant models. This proof-of-concept study opens the possibility to target one of the most incurable forms of AML with specific EVI1-CTBP inhibitors. This has important implications for other tumor types with aberrant expression of EVI1 and for cancers transformed by different CTBP-dependent oncogenic transcription factors.

## Teaser

Incurable leukemias driven by the EVI1 oncoprotein can be reversed experimentally by blocking the binding to its cofactor CTBP2.

## INTRODUCTION

Aberrant activation of *EVI1* in acute myeloid leukemia (AML) is associated with poor treatment response and diminished survival (*1*). *EVI1*-expressing leukemias include those with 3q26/*MECOM* (*MDS1* and *EVI1* complex locus, from which *EVI1* is transcribed) or 11q23/*MLL* rearrangements. In the case of 3q26/*MECOM* rearrangements, active enhancers translocate toward the *MECOM* locus (*2*–*5*). In approximately 40% of 11q23/*MLL*-rearranged patients with AML, *EVI1* is overexpressed by a mechanism that is incompletely understood (*6*). Irrespective of the mechanism of activation, EVI1 is essential for the survival, proliferation, and the undifferentiated phenotype of those AML cells (*3*). Consequently, eliminating EVI1 or interfering with its function may constitute an effective therapeutic strategy for these aggressive forms of AML.

*EVI1* encodes a nuclear protein containing two DNA binding zinc-finger domains, which is an essential transcriptional regulator that has important repressive functions (*7*, *8*). In addition to *EVI1*, a long isoform *MDS1*-*EVI1* (*PRDM3*) is also transcribed from this locus. The key difference between these two isoforms is that *MDS-EVI1* contains a SET domain implicated in histone methylation (*9*). However, *MDS1-EVI1* is not or hardly expressed in 3q26/*MECOM*-rearranged AML (*2*). This raises the question of how EVI1 represses transcription and causes leukemic transformation. It is likely that EVI1 recruits other proteins to its binding sites in the genome to exert its repressive effect. Exact knowledge about those partners and the mechanism of interaction may be used to target EVI1-driven AML.

Several EVI1 interaction partners have been identified previously in various experimental systems, including CTBP1/2, MBD3, EHMT2, HDAC1/2, SUV39H1, and SMAD3 (*10*–*16*). In this study, we investigated which proteins bind to endogenous EVI1 in AML cells and are essential for leukemic transformation. We identified CTBP1 and CTBP2 as the most enriched EVI1-binding partners and showed that the interaction between EVI1 and CTBP2 is essential for leukemic cell proliferation. This interaction depends on a PLDLS domain within EVI1. A PLDLS-containing repeat construct outcompetes EVI1-CTBP interaction and reverses the leukemic phenotype. This proof-of-concept study demonstrates the possibility of targeting a subset of high-risk AML by interfering between EVI1 and its protein partners.

## RESULTS

### EVI1 is essential for CTBP2 recruitment to chromatin in inv(3) AML

**Fig. 1.**
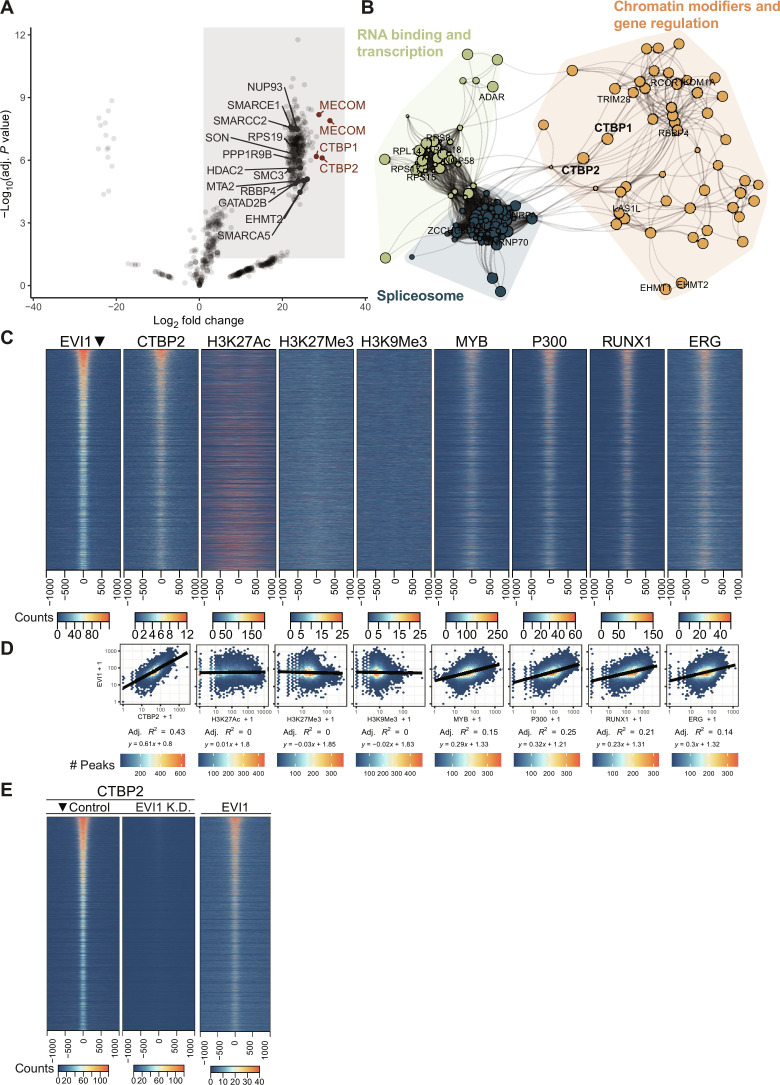
CTBP1 and CTBP2 bind EVI1. (**A**) Differential enrichment of proteins binding to EVI1 as determined by an EVI1 specific IP compared to a nonspecific IgG IP followed by MS in MUTZ3 nuclear protein extracts (*n* = 3 for each group, thresholds log_2_FC > 1 and *P* value < 0.05). FC, fold change. Top 4 enriched gene identifiers are highlighted in red [MECOM (EVI1), CTBP1, and CTBP2]. In addition, several previously identified MECOM interactors are shown in gray (BioGrid). (**B**) Network plot of protein complex enrichment analysis of the proteins coprecipitated with EVI1 IP in MUTZ3 cells. The connections (edges) between proteins (nodes) were weighted on the basis of their co-occurrence in CORUM complexes. Node color represents the cluster assigned to a protein; dot size represents the fold enrichment in the IP. Font size of CTBP1 and CTBP2 was adjusted manually, and colored areas and their corresponding labels were added manually based on the common biochemical functions. The full plot with all labels is presented in fig. S1C. (**C**) Heatmaps of ChIP-seq experiments in MUTZ3 cells using antibodies directed to the indicated transcription factors or histone modifications. Ranking was based on the ChIP-seq EVI1 signal (leftmost panel) showing signal intensity of indicated ChIP-seq tracks in a ± 1000-bp region centered on EVI1 peaks (21505 peaks). (**D**) Quantification of the heatmap in (C). Normalized EVI1 signal is plotted versus the normalized reads in the indicated ChIP-seq tracks for all EVI1 peaks with a window of ±1000 bp. Correlation coefficients and linear regression equations are shown for log_10_-transformed data with a pseudo count of 1. (**E**) Heatmap of CTBP2 ChIP-seq data following dox-inducible short hairpin RNA–mediated knockdown of *EVI1* (48 hours) in MOLM1 cells. On the right, EVI1 heatmap in unperturbed MOLM1 cells. Tracks are ranked on peaks in the CTBP2 control track (leftmost panel, 64,569 peaks).

EVI1 has been reported to interact with a wide range of repressive transcriptional and epigenetic regulators, but it is unclear which of those are consistently required for EVI1-mediated transformation. To identify EVI1-binding proteins in 3q26/*MECOM*-rearranged AML, we performed EVI1 immunoprecipitation (IP) followed by mass spectrometry (MS) in nuclear lysates (EVI1-IP/MS) from MUTZ3 cells. We detected several EVI1 binding partners previously reported on BioGRID, including CTBP1/2, HDAC2, RBBP4, MTA2, and EHMT2, of which CTBP1 and CTBP2 were most enriched compared to immunoglobulin G (IgG) control (Fig. 1A) (*10*–*12*, *16*–*19*). We confirmed the reciprocal interaction by EVI1- or CTBP2-IP, followed by Western blot (WB) for CTBP2 (EVI1-IP/CTBP2-WB) or EVI1 (CTBP2-IP/EVI1-WB), respectively (fig. S1A). In addition, we could also detect EVI1-CTBP2 interaction in an inv(3) patient sample (fig. S1B). We performed enrichment analysis with EnrichR on the 460 significantly co-immunoprecipitated proteins using protein complexes from the comprehensive resource of mammalian protein complexes (CORUM) database (Fig. 1B and fig. S1C) (*20*). CTBP1 and CTBP2 have a central position in the network visualization of enriched proteins connected and clustered by their co-occurrence in protein complexes (Fig. 1B and fig. S1C). To validate our findings in a different model, we performed streptavidin-based protein precipitation on murine EVI1 fused to a biotag biotinylated in vivo by *Escherichia coli* biotin ligase BirA, in the murine leukemia cell line NFS78 (fig. S1D) (*21*). Here, we again identified CTBP1/2 as most enriched compared to IgG control (fig. S1E). Because CTBP1 and CTBP2 are highly homologous at protein level and both equally coprecipitated with EVI1, we decided to focus on CTBP2 to represent EV1-CTBP interaction. Publicly available data support this choice. Functional redundancy between CTBP1 and CTBP2 is supported by the Cancer Dependency Map (DEPMAP) database. All cancer cell lines studied in this dataset express CTBP1, and the majority also express CTBP2 (fig. S2A). Cell lines are more dependent on CTBP1 if they do not express CTBP2, while simultaneous dependency on both is rare (fig. S2, B and C). This is also true for the subset of hematological cell lines in DEPMAP, although none of these are dependent on EVI1 (fig. S2, D to F). This suggests a general redundancy between CTBP1 and CTBP2. This is also observed in a paralog screen in HAP1 cells (*22*) (fig. S2G). In contrast, in EVI1-expressing HNT34 cells, codependency of CTBP2 and EVI1 has been reported (fig. S2H) (*23*). Chromosome 3q26–rearranged EVI1^+^ SB1690CB cells show significant growth disadvantage when CTBP2 is knocked down (fig. S2, I and J). Chromatin immunoprecipitation sequencing (ChIP-seq) using chromatin from MUTZ3 cells (Fig. 1C) showed a strong reciprocal correlation between EVI1 and CTBP2 binding sites (Fig. 1D and fig. S3, A and B). This was much higher than the correlation of either EVI1 or CTBP2 with factors such as p300, MYB, or RUNX1 (Fig. 1D and fig. S3A). Furthermore, knockdown of *EVI1* (fig. S3C) resulted in a complete loss of CTBP2 signal in ChIP-seq (Fig. 1E), demonstrating that EVI1 is critical for CTBP2 to engage with chromatin in 3q26-rearranged cells.

### A single PLDLS domain in EVI1 is critical for CTBP2 binding

**Fig. 2.**
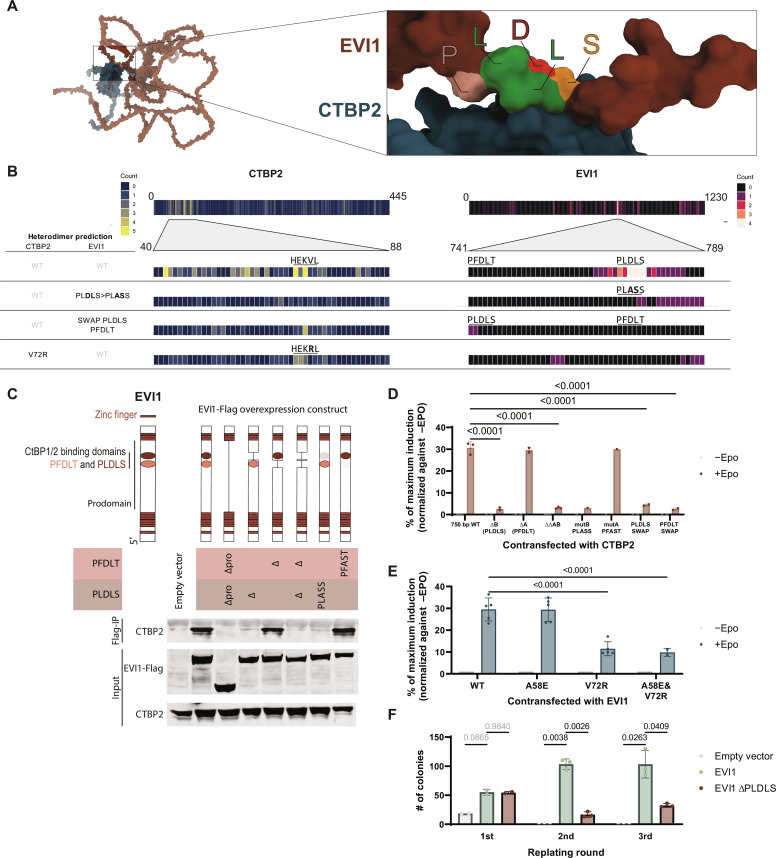
Identification of a competitive inhibitor of EVI1-CTBP2 interaction. (**A**) EVI1-CTBP2 interaction as predicted by AlphaFold and visualized with ChimeraX. EVI1 is shown in maroon, with the PLDLS residues highlighted. CTBP2 is depicted in dark blue. (**B**) Frequency of the involvement of individual residues in EVI1 and CTBP2 in the interaction between these two proteins as predicted by AlphaFold and quantified in ChimeraX (using the “interfaces” command with default parameters) for all 25 structures per heterodimer prediction. (**C**) IP of overexpressed EVI1-FLAG with anti-FLAG antibody, followed by WB for CTBP2 and FLAG in lysates from HEK293T cells transfected with wild type (WT) or mutated *EVI1*-FLAG. The diagram indicates sites of deletions and mutations. (**D**) MAPPIT assay in HEK293T cells to measure EVI1-CTBP interaction. Mutations introduced in EVI1 are similar to (C). Reporter induction is normalized to –EPO (not induced) and maximum induction with RNF as positive control. Statistical significance is determined with a mixed-effects model (significance shown if *P* < 0.05 and *N* > 1) in one to three independent experiments (all independent experiments are performed in triplicate); means + SD is shown. (**E**) MAPPIT assay in HEK293T cells to measure EVI1-CTBP2 interaction, with mutations disrupting residues in CTBP2. Those residues have been previously reported to be important for target protein binding via PXDLS motifs (*n* = 2 to 5 in triplicate) (*53*). Reporter induction is normalized to –EPO (not induced) and maximum induction with RNF as positive control. Statistical significance is determined with a two-way analysis of variance (ANOVA) (significance shown if *P* < 0.05) in two to five independent experiments (all independent experiments are performed in triplicate); means + SD is shown. (**F**) Colony formation of mouse bone marrow cells transduced with empty vector, full-length *EVI1*, or *EVI1* without PLDLS domain. Statistical significance was determined with a two-way ANOVA with EVI1 as reference (all comparisons shown). Experiment performed in triplicate.

To study whether the interaction between EVI1 and CTBP2 is required for transformation, we first set out to determine which amino acids in EVI1 bind CTBP2. Computational modeling of protein-protein interfaces with AlphaFold (*24*) predicted interaction between EVI1 and CTBP2 via a PLDLS site in EVI1 (Fig. 2, A and B) with R43, H69, and K71 in CTBP2 (Fig. 2B). In line with this prediction, CTBP proteins have been reported to bind to PXDLX motifs, of which there are two in EVI1, i.e., a PFDLT and a PLDLS motif (Fig. 2, B and C) (*25*). To experimentally determine whether it is indeed the PLDLS site in EVI1 that interacts with CTBP2, distinct FLAG-tagged EVI1 mutants with deletions of PFDLT, PLDLS, or both were introduced into human embryonic kidney (HEK) 293T cells. Upon FLAG-IP, only the proteins with an intact PLDLS site were able to bind CTBP2 (Fig. 2C). In addition, FLAG-tagged full-length EVI1 in which PLDLS was mutated into PLASS also lost CTBP2 interaction (Fig. 2C). This is in line with a reduced interaction frequency predicted by AlphaFold for a heterodimer of CTBP2 and PLASS-mutant EVI1 (Fig. 2B). An EVI1 construct in which PFDLT was mutated into PFAST was still able to bind CTBP2, confirming that only PLDLS is essential for CTBP2 binding (Fig. 2C).

To enable rapid and direct measurement of interaction between EVI1 and CTBP2 and to study the effect of different mutations, we adapted and applied the luciferase-based Mammalian protein-protein interaction trap (MAPPIT) assay (fig. S4A) (*26*). In short, this assay uses Janus kinase (JAK)–signal transducers and activators of transcription (STAT) signaling downstream of a modified erythropoietin (EPO) receptor to quantify protein-protein interaction. EPO can activate a STAT3-responsive luciferase reporter only if there is interaction between bait and prey proteins, in this case EVI1 and CTBP2 (fig. S4A). In the MAPPIT assay, the target protein-protein interaction takes place in the cytosol, which means that most other nuclear proteins are absent, and any interaction measured is likely to occur directly between the two studied proteins. With this assay, we confirmed that only by deleting or mutating the PLDLS site the luciferase activity was abolished (Fig. 2D). In addition, moving the PLDLS site to the PFDLT location and vice versa was not sufficient to rescue the interaction between EVI1 and CTBP2. This is in line with predictions by AlphaFold (Fig. 2, B and D). These results indicate that the PLDLS residues themselves, as well as some surrounding amino acids, are likely essential for the interaction between EVI1 and CTBP2. Within CTBP2, EVI1 is predicted to interact mostly with the N-terminal region, with a focus point on H69 and K71, which are part of a small α helix (Fig. 2B). The valine residue seems critical for maintenance of this helix; mutating H69A and K71A directly had a modest effect on the interaction between EVI1 and CTBP2 as determined by MAPPIT (fig. S4B). Reduced EVI1-CTBP2 interaction was predicted with AlphaFold when adjacent residue V72 in CTBP2 was mutated into an arginine (V72R) (Fig. 2B). Indeed, using MAPPIT, we determined that in CTBP2, the valine residue at position 72 (V72) was important for binding EVI1, whereas alanine at position 58 (A58) was not (Fig. 2E). In summary, with its PLDLS site, EVI1 recognizes a specific HEKV binding region in CTBP2.

The importance of the PLDLS motif in EVI1-mediated transformation was studied in mouse bone marrow progenitors in vitro. Transduction with a retroviral construct carrying wild-type *Evi1* formed colonies of cells that can be replated indefinitely (Fig. 2F). Serial replating capacity of mouse bone marrow cells transduced with mouse *Evi1* lacking the PLDLS domain was severely reduced, demonstrating the importance of this specific domain and its interactor CTBP2 for EVI1-mediated transformation of bone marrow progenitors (Fig. 2F).

### Overexpression of a PLDLS competitor causes loss of EVI1 to CTBP2 interaction

**Fig. 3.**
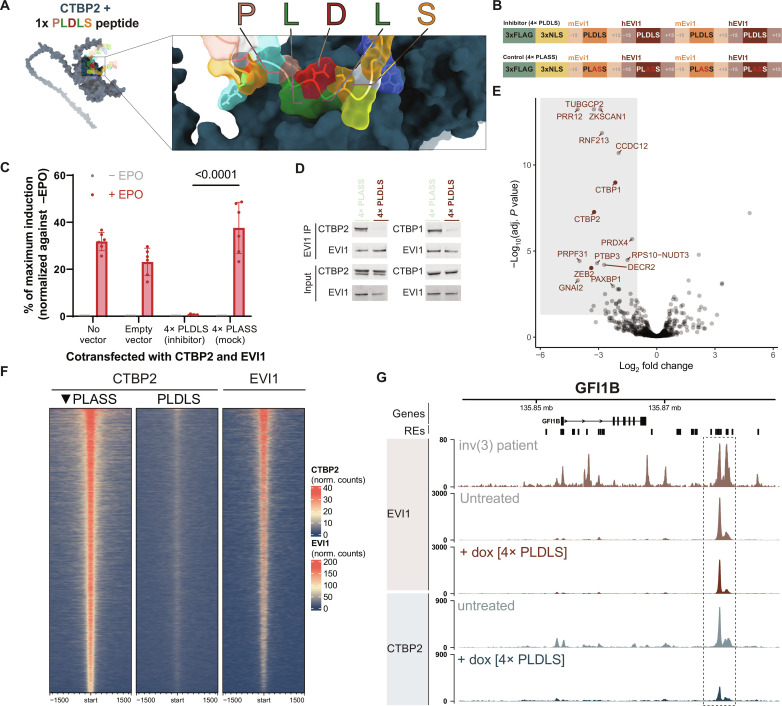
Disruption of CTBP2 binding to EVI1 with PLDLS inhibitor. (**A**) AlphaFold prediction of the interaction of the PLDLS inhibitor with CTBP2 visualized with ChimeraX. The PLDLS residues are shown in different colors according to their biochemical properties and CTBP2 is depicted in dark blue. (**B**) Diagram outlining the 4× PLDLS inhibitor and 4× PLASS control constructs. Each repeat consists of two human (hEVI1) and two mouse (mEvi1) PLDLS surrounding region (±15 amino acids); NLS, nuclear localization signal. (**C**) MAPPIT assay in HEK293T cells to measure EVI1-CTBP2 interaction as in Fig. 2 with 4× PLDLS inhibitor or 4× PLASS control. Reporter induction is normalized to –EPO (not induced) and maximum induction with RNF as positive control. Significance was determined with two-way ANOVA (comparison PLDLS versus PLASS + EPO shown). (**D**) WB for CTBP1 and CTBP2 following IP for EVI1 in MUTZ3 cells with dox-induced 4× PLASS or 4× PLDLS expression. (**E**) Differential enrichment in MS following IP for EVI1 in MUTZ3 cells with dox-induced 4× PLDLS or 4× PLASS expression. A negative log_2_ fold change indicates depletion from EVI1 complex upon PLDLS overexpression; the proteins that fall into the gray rectangle have reduced binding to EVI1 upon overexpression of 4× PLDLS. (**F**) Heatmap of ChIP-seq data for CTBP2 in MUTZ3 cells following retroviral transduction with either 4× PLASS (control) or 4× PLDLS (inhibitor). Consensus CTBP2 binding sites were determined by DiffBind (5634 peaks). Tracks were ranked by the average DiffBind enrichment in the control tracks. Enrichment in an untreated EVI1 track is shown for comparison. (**G**) Representative plot showing the effect of 4× PLDLS overexpression induction with doxycycline (72 hours) on the locus of *GFI1B*. (RE, regulatory element associated with expression of *GFI1B*). Top track is on primary material from an inv(3) patient; the other tracks are from MUTZ3.

Next, we wondered whether it is possible to interfere between EVI1 and CTBP2 using a competing PLDLS-containing construct. First, we determined the minimal region around PLDLS required for a competitor peptide to bind CTBP2. An EVI1-PLDLS construct with 10 to 15 amino acids at either side of PLDLS fully induced the STAT3-driven luciferase activity in the MAPPIT assay with CTBP2 as prey protein (fig. S4C). With AlphaFold, a 1x PLDLS repeat was predicted to bind CTBP2 at the same site where full-length EVI1 binds (Fig. 3A). We next designed a construct with four repeats of the PLDLS motif including 15 flanking amino acids from EVI1 on either side of each PLDLS site (Fig. 3B). The 4× PLDLS construct was able to fully outcompete the EVI1/CTBP2 interaction in the MAPPIT assay, whereas a similar 4× PLASS control did not affect EVI1/CTBP2 interaction (Fig. 3C). In line with this, EVI1-IP/CTBP2-WB demonstrated loss of CTBP2 binding to EVI1 in the presence of 4× PLDLS, whereas 4× PLASS construct did not affect EVI1-CTBP2 interaction in MUTZ3 cells (Fig. 3D and fig. S4E). The interaction between EVI1 and CTBP1 was also specifically outcompeted by 4× PLDLS, indicating that both proteins can bind EVI1 via the same motif (Fig. 3D). MS-IP of EVI1 showed loss of CTBP1, CTBP2, and ZEB2, among a handful of other proteins from the EVI1 complex (Fig. 3E). ZEB2 is another one of the 41 proteins in the human proteome that contain a PLDLS site (fig. S5A). Apart from the EVI1-CTBP2 interaction, ZEB2-CTBP1/2 may therefore also be affected with this 4× PLDLS construct. Of the human PLDLS-containing proteins, only three more are detected in nuclear lysates of MUTZ3 (ZEB1, BCOR, and DGKA) (fig. S5B). The PLDLS site of DGKA was not predicted to be able to interact with CTBP2 by AlphaFold (fig. S5C). On the basis of the clustering analysis, ZEB1/2 and BCOR have a relatively distinct PLDLS amino acid context from MECOM. Using MAPPIT, we demonstrate that ZEB2-CTBP2 interaction can be outcompeted by overexpression of the PLDLS inhibitor (fig. S4D). However, as demonstrated in Fig. 1E, elimination of EVI1 causes loss of CTBP2 binding to chromatin. CTBP2 association to chromatin is not taken over by other PLDLS containing proteins such as ZEB1/2 in these cells.

Upon transduction of MUTZ3 cells with 4× PLDLS, CTBP2 binding to chromatin was globally lost, whereas 4× PLASS control did not affect CTBP2 interaction with chromatin (Fig. 3, F and G and fig. S6A). In addition, EVI1 binding to chromatin was only mildly affected by overexpression of 4× PLDLS (fig. S6B). This shows that in 3q26/*MECOM*-rearranged leukemia cells, the effect of overexpression of the 4× PLDLS inhibitor on CTBP2 binding to chromatin is similar to *EVI1* knockdown (Fig. 1E). In addition, while down-regulation of CTBP2 binding to chromatin was global, it was even stronger in CTBP2 peaks that overlap with an EVI1 binding site in ChIP-seq for MUTZ3 (fig. S6C). We next studied the effect of 4× PLDLS overexpression on the transcriptome of MUTZ3 cells. We identified 162 significantly differentially expressed genes (126 up-regulated and 36 down-regulated), of which 70% of up-regulated and 50% of down-regulated genes contained at least one CTBP2 binding site in their locus (fig. S6D). Twenty-nine of the top 30 CTBP2 peaks that are annotated to 4× PLDLS target genes are up-regulated genes in RNA sequencing (RNA-seq), in line with a repressor function of EVI1-CTBP2 (fig. S6E).

### The PLDLS competitor abolishes leukemic potential of EVI1-transformed mouse bone marrow cells

**Fig. 4.**
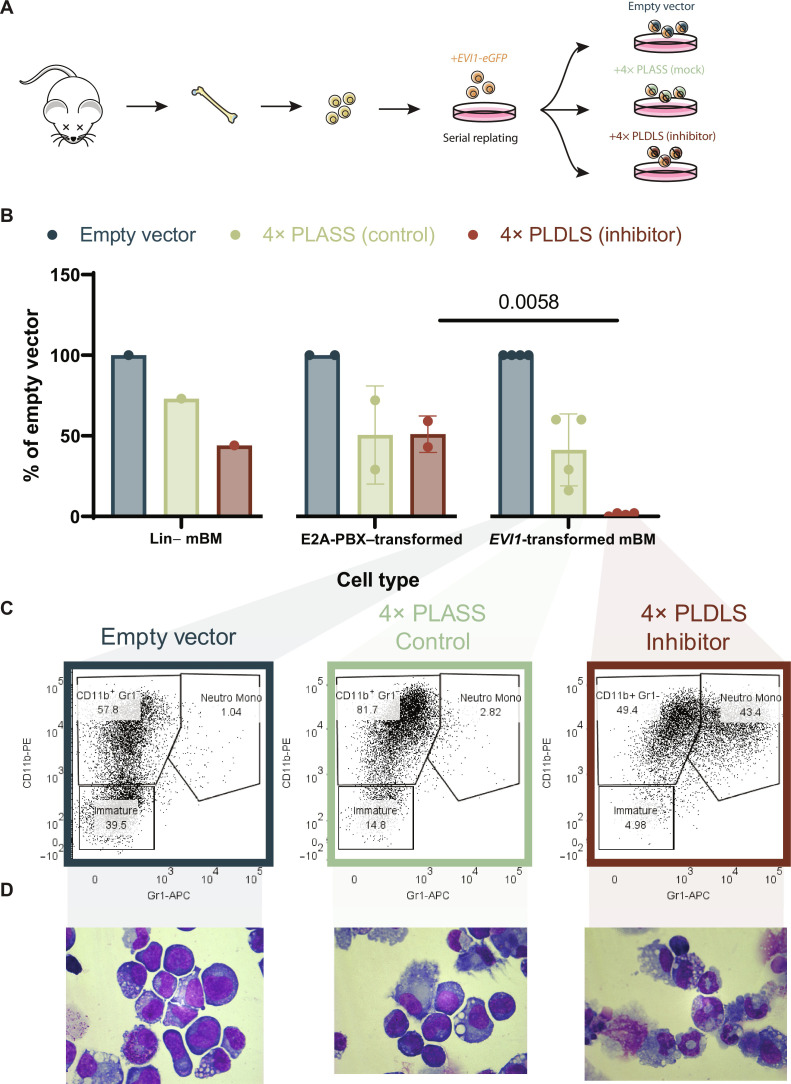
Inhibition of EVI1-CTBP2 in transformed mouse bone marrow abolishes leukemic potential. (**A**) Diagram illustrating the procedure for functionally testing inhibitors of the EVI1-CTBP interaction. For all experiments on *EVI1*-transformed bone marrow shown, bone marrow of 8- to 10-week-old mice was collected and transduced with a retroviral *EVI1*-*eGFP* construct. The immortalized cells acquired after four rounds of serial replating were transduced with retroviral vectors containing no insert (empty vector), a 4× PLASS control, or 4× PLDLS (inhibitor). (**B**) Colony formation of lineage-negative (Lin^−^) mouse bone marrow cells (left), Lin^−^ cells immortalized with *E2A-PBX* (middle), or with *EVI1* (right). Before plating, cells were transfected with empty vector, PLASS (control), or PLDLS (inhibitor) and selected with puromycin. All experiments were performed in triplicate; each dot represents the average of technical triplicates of an independent experiment (*n* = 1 to 4). Adjusted *P* value of two-way ANOVA with Šídák’s multiple comparisons test shown if adjusted *P* value < 0.05. Between-group comparison shown on graph; within-group test results are included in table S1. (means + SD plotted). (**C**) Flow cytometric analysis of Lin^−^ mouse bone marrow transduced with *EVI1*, and with empty vector, 4× PLASS, or 4× PLDLS, stained with CD11b-phycoerythrin (PE) and Gr1-allophycocyanin (APC) (single-cell gate shown) [day 7; cultured in media with cytokines including granulocyte-macrophage colony-stimulating factor (GM-CSF)]. (**D**) May-Grünwald Giemsa staining of cytospins made from Lin^−^ mouse bone marrow transduced with *EVI1*, together with empty vector, 4× PLASS, or 4× PLDLS (day 7; cultured in media with cytokines including GM-CSF) [same experiment as (C); magnification, ×63].

We next investigated whether interference between EVI1 and CTBP2 using by 4×-PLDLS affects *Evi1*-driven transformation in mouse bone marrow cells (Fig. 4A). Colony assays demonstrated that the replating ability of *Evi1*-transformed bone marrow was fully abolished when these cells were transduced with a 4× PLDLS competitive inhibitor, whereas the 4× PLASS mock inhibitor had no effect (Fig. 4B and table S1). In *E2a-Pbx* transformed bone marrow, the effect of 4× PLDLS on colony formation was significantly smaller than in *Evi1*-transformed bone marrow (Fig. 4B). In the presence of 4× PLDLS, but not 4× PLASS, we observed strong neutrophilic differentiation of the *Evi1*-transformed bone marrow cells determined by flow cytometric analysis (Gr1^+^/CD11b^+^) and by May-Grünwald Giemsa staining of cytospins (Fig. 4, C and D, and fig. S7A). Those experiments indicate that the differentiation arrest of myeloid progenitors caused by *Evi1* overexpression depends on the interaction with CTBP1/2. Together, these results indicate that *Evi1*-transformed cells are unable to sustain their leukemic potential in the presence of the PLDLS inhibitor.

### The PLDLS inhibitor blocks in vivo outgrowth of human EVI1-transformed AML cells

**Fig. 5.**
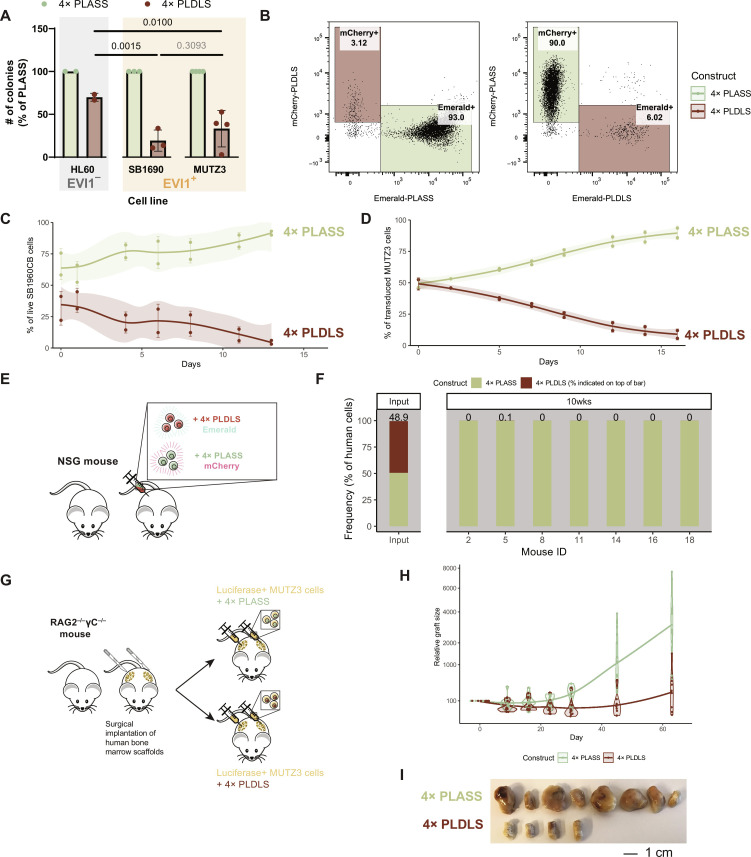
In vivo growth inhibition of EVI1-transformed human leukemia cells using EVI1-CTBP2 inhibitor. (**A**) Colony formation of human *EVI1*^−^ (HL-60) or *EVI1*^+^ (SB1690 or MUTZ3) cell lines, which are retrovirally transduced with either 4× PLDLS or 4× PLASS. All experiments were performed in triplicate, *n* = 2 to 4 experiments per cell line (two-way ANOVA with multiple testing correction; within-group test results in table S1). (**B**) SB1690 cells were lentivirally transduced and mixed to 1:1 ratio Emerald:mCherry on day 0. Flow cytometric analysis at day 13 is shown. Left: 4× PLDLS-mCherry and 4× PLASS-Emerald; right: 4× PLDLS-Emerald and 4× PLASS-mCherry. (**C**) Competition between 4× PLASS and 4× PLDLS in SB1690CB over time. Frequencies are grouped by whether they contain inhibitor (PLDLS) or control (PLASS). (**D**) Competition between 4× PLASS and 4× PLDLS in MUTZ3 over time. Quantification of Emerald and mCherry gates as a fraction of all transduced cells in the experiment (fig. S7B) grouped by whether they contain inhibitor (PLDLS) or control (PLASS). (**E**) Outline of SB1690CB competition in NSG mice. Before transplant, cells were separately transduced with PLDLS-Emerald or PLASS-mCherry and mixed in a 1:1 ratio. (**F**) Flow cytometric analysis of input and bone marrow of seven mice transplanted with SB1690. Percentage of 4× PLDLS-positive cells is indicated above each bar. (**G**) Diagram of procedure to engraft human MUTZ3 into NSG mice. Before transplantation, human bone marrow scaffolds are surgically implanted. Mice are injected with either MUTZ3-Luciferase-4× PLDLS inhibitor or MUTZ3-Luciferase-4× PLASS control. Upon injection with luciferin, mice are imaged in a luminescence scanner to monitor outgrowth (fig. S7E). (**H**) Tumor area of mice who received either a transplant of MUTZ3-Luciferase-4× PLDLS inhibitor or MUTZ3-Luciferase-4× PLASS control (*n* = 4 mice with two scaffolds each for PLDLS and *n* = 6 mice with two scaffolds each for PLASS). (**I**) Scaffolds of mice with engraftment of 4× PLASS or 4× PLDLS.

Next, we investigated the effect of the 4× PLDLS inhibitor on EVI1-transformed human leukemia cells. Overexpression of 4× PLDLS strongly reduced in vitro colony formation of the *EVI1*-positive 3q26-rearranged AML cell lines MUTZ3 and SB1690CB compared to colony formation in the presence of 4× PLASS (Fig. 5A and table S1). While there is a partial effect of 4× PLASS versus empty vector, this may be due to the high protein production from the retroviral construct. The effect was much stronger than in EVI1-negative cell line HL60 (Fig. 5A). To measure the direct competition between cells expressing either 4× PLASS or 4× PLDLS, we transduced SB1690CB and MUTZ3 cells with either PLDLS- or PLASS-containing vectors. To enable cell sorting and tracking over time, each vector contained a fluorescent read out (mCherry or Emerald). At day 0, cells were sorted and mixed in approximately 1:1 ratio of PLDLS- and PLASS-containing fractions. Two replicate cell line mixtures were generated: PLDLS-mCherry/PLASS-Emerald and PLDLS-Emerald/PLASS-mCherry. PLASS-containing cells always outcompeted PLDLS-transduced cells in vitro in both SB1690CB (Fig. 5, B and C) and MUTZ3 (Fig. 5D and fig. S7B). The effect of the 4× PLDLS inhibitor on the outgrowth of EVI1-expressing AML cells in vivo was studied in two xenotransplant models. First, the mCherry^+^ and Emerald^+^ mixtures of SB1690CB cell were injected into six mice in a 1:1 ratio (Fig. 5E). Flow cytometric analysis at input showed the equal distribution of mCherry^+^ and Emerald^+^ cells at the start of the experiment, whereas after 10 weeks, virtually, only PLASS-containing cells were detectable (Fig. 5E, fig. S7, C and D). In a second in vivo model, we transplanted human MUTZ3 cells into NOD *SCID* Gamma (NSG) mice with surgically implanted human bone marrow scaffolds (Fig. 5G) (*27*, *28*). The MUTZ3 cells transplanted into these mice contained a cytomegalovirus promoter-driven luciferase construct, enabling live measurement of graft size over time. Outgrowth of MUTZ3-luciferase cells with 4× PLDLS inhibitor was strongly reduced compared to 4× PLASS control cells (Fig. 5, A and H, and fig. S7E). Tumors that were detectable at the implanted scaffolds in mice transplanted with 4× PLDLS-containing cells were smaller than those in scaffolds with 4× PLASS-containing MUTZ3 cells (Fig. 5I). Thus, human *EVI1*-transformed AML cells depend on the PLDLS-dependent interaction between CTBP and EVI1, which can be targeted to reduce the growth of this leukemia in vivo.

## DISCUSSION

Patients with AML with overexpression of *EVI1* caused by inv(3) or t(3;3) have an extremely poor prognosis and are frequently refractory to current treatments (*1*). Consequently, there is a major need for the development of new ways to treat these patients. We have previously demonstrated that the survival and growth of those leukemia cells heavily depend on *EVI1* (*3*). Here, we show that EVI1 interacts with CTBP1/2, that the interaction is essential for leukemic transformation, and that it can be targeted. The interaction between EVI1 and CTBP1/2 has previously been reported (*10*, *25*), and also recently in hematopoietic cell lines (*29*), but its importance in AML development was not shown. We found that in AML cells, CTBP2 predominantly interacts with EVI1 via a conserved PLDLS motif, which was previously described to mediate interaction with CTBP1 in rat fibroblasts (*25*). We demonstrate here that without this PLDLS motif or following the overexpression of a PLDLS competitor construct, transformation by *EVI1* in 3q26/*MECOM*-rearranged AML is abolished.

A key question that remains is whether inhibition via a PLDLS domain (which is present in more proteins than EVI1) specifically targets EVI1, and whether EVI1-expressing cells are uniquely sensitive to this type of inhibition. CTBP2 interacts with ZEB2, and using the MAPPIT assay, we showed that the PLDLS inhibitor affects this interaction as well (fig. S4D). Likewise, CTBP2 can interact with E1A, AREB6, or FOG (*30*). In addition, it has been shown that E1A and CTBP1 can be outcompeted with an E1A mimicking peptide as well (*31*). However, we found global loss of CTBP2 binding to chromatin upon EVI1 knockdown or when the 4× PLDLS inhibitor was overexpressed. This indicates that EVI1 is critical for CTBP2 binding in 3q26/*MECOM*-rearranged AML cells and that ZEB2 or other PLDLS containing proteins present in the cells are unable to functionally replace EVI1 in tethering CTBP2 to chromatin. In addition, we demonstrate less sensitivity to PLDLS inhibition in HL60 cells, mouse bone marrow progenitors, and E2A-PBX–transformed mouse bone marrow, none of which express EVI1. Thus, although other proteins might be affected by inhibition by 4× PLDLS, the fact that EVI1-expressing cells are more sensitive to the effect of PLDLS inhibition suggests that there is a therapeutic window for targeting this interaction in AML patients.

It is unclear what the biological role of CTBP1/2 is in the transformation process. Perhaps, their only function is to serve as scaffold proteins that bring other critical proteins to various specialized complexes. While CTBP1 and CTBP2 are highly enriched in MS-IP for EVI1 and important for EVI1 function, many other proteins interact with EVI1 and may also play a role in transformation. For instance, we identified HDAC1/2, RCOR1, RUNX1, and EHMT1/2 among ~400 significant proteins in our IP-MS experiments, which are known interactors of CTBP1/2. In addition, there might also be important interactions of EVI1 that are not mediated by CTBP1/2. The importance of CTBP-dependent or CTBP-independent EVI1 interactors in leukemic transformation should be further investigated using a genetic knockout screen. It is also possible that there is an additional role for CTBPs in modulating chromatin bound by EVI1. CTBP1 and CTBP2 are highly structured proteins that contain two large oxireductase enzymatic domains (*32*). Both are annotated as catalysts of a nicotinamide adenine dinucleotide (NAD+) to reduced form of nicotinamide adenine dinucleotide phosphate (NADPH) conversion in the methionine salvage pathway, although this has been studied mostly for CTBP1 (*33*–*35*). Nicotinamide adenine dinucleotide (oxidized form) (NAD) binding has been reported to be important for homo- or heterodimerization of CTBPs with each other (*36*). It is unclear what substrate CTBP1/2 catalyze while in complex with EVI1, and whether the product of this chemical reaction is limiting for any other metabolic reaction taking place at chromatin. It would be interesting to investigate the effect of mutating enzymatic domains in CTBP1/2 on transformation by EVI1. Metabolic profiling of nuclei, perhaps incorporating spatial information in the presence of our PLDLS inhibitor, could reveal whether CTBP1/2 has any local effects on chromatin that might be important for gene regulation.

Together, in this proof-of-concept study, we show that interference with protein-protein interfaces of transcription factors such as EVI1 and their cofactors can inhibit tumor growth and should be exploited for therapeutic purposes. Because *EVI1* overexpression has also been reported in other tumor types, such as ovarian or breast cancer, our findings may not be restricted to 3q26/*MECOM-*rearranged AMLs. Whether PLDLS-to-CTBP interaction is essential in those tumors needs to be investigated. Generation of chemically optimized EVI1-derived PLDLS peptides might lead to the development of stable small molecules that interfere with EVI1 and CTBP1/2 complex formation. The effect of these compounds on EVI1-driven leukemia and healthy HSPCs should be tested in vivo. These studies will also reveal the role of CTBP1/2 in normal hematopoesis. Ultimately, clinical testing may pave the way toward a new treatment option for this highly aggressive form of AML caused by overexpression of *EVI1*.

## MATERIALS AND METHODS

### Data availability

ChIP-seq data are made available on Gene Expression Omnibus (GEO) under accession number GSE236010. Raw MS data and data for protein identification and quantification are submitted to the ProteomeXchange Consortium via the PRIDE partner repository with the data identifier PXD043333 [MUTZ3 wild type (WT) IP] and PXD048760 (MUTZ3 PLDLS versus PLASS IP). All other data used for generating figures presented here (AlphaFold predicted structures, flow cytometry frequencies, uncropped WBs, luciferase output files, and MS clustering and quantification) are available at Zenodo under accession https://zenodo.org/records/10650341. Custom scripts for AlphaFold quantifications, ChIP-seq correlation plots, and protein alignment of PLDLS sites in human proteome are available on Github (https://github.com/dorienpastoors/EVI1_CTBP2_PLDLSinhibitor). An overview of all data deposited associated with this manuscript and in which figure panels are used is available in table S1.

### Cell lines

MUTZ3 (DSMZ, catalog no. ACC295) cells were cultured in Alpha mem medium (Gibco, catalog no. 2571038) supplemented with 20% supernatant of urinary bladder carcinoma cell line 5637 (DSMZ, catalog no. ACC35) (*37*), and 20% fetal calf serum (FCS) (500 ml; Sigma-Aldrich, catalog no. F7524). SB1690CB cells were cultured in RPMI (Gibco, catalog no. 21875-034), 10% supernatant 5637, 10% FCS, and human interleukin-3 (IL-3) (10 ng/ml; PeproTech, catalog no. 200-03). MOLM1, HNT34, and HL60 (DSMZ, catalog no. ACC720, ACC600, and ACC3) were cultured in RPMI and 10% FCS. 293 T (DSMZ, catalog no. ACC635) cells were cultured in Dulbecco’s modified Eagle’s medium (DMEM) (Gibco, catalog no. 2430-025) and 10% FCS. All medium used was supplemented with penicillin (50 U/ml) and streptomycin (50 U/ml; Gibco, catalog no. 15140122). All cell lines were routinely confirmed to be mycoplasma free by using the MycoAlert Mycoplasma Detection kit (Lonza, catalog no. LT07-318). SB1690CB was a gift of S. Meyer (*38*).

### Generation of viral supernatants

Retroviral supernatants were prepared by cotransfecting 293T cells (DSMZ, catalog no. ACC635) using pCL ECO (Addgene, catalog no. 12371) for mouse cells or pCL ampho for human cells and a retroviral vector. Retrovirus was harvested at 48 hours after transfection. Lentiviruses were produced by cotransfecting 293T cells using psPAX2 (Addgene, catalog no. 12260), pMD2.G (Addgene, catalog no. 12259), and a lentiviral vector. Lentiviral supernatants were harvested at 72 hours after transfection. All transfections were performed using Fugene-6 transfection reagent according to the manufacturer’s protocol (Promega, catalog no. E2691).

### Overexpression EVI1 / PLDLS EVI1 mutant in murine HCS and colony forming assays

Eight- to 10-week-old female C57bl/6 mice (the Jackson Laboratory) were euthanized, and bone marrow was isolated from their femurs and tibias. Erythrocytes were lysed by resuspending total bone marrow in ACK-lysing solution (Gibco, catalog no. A10492-01). Mononucleated cells were enriched for hematopoietic progenitor stem cells following manufacturers protocol (BD, catalog no. 558451). After isolation, cells were cultured overnight in CellGro SCGM medium (CellGenix, catalog no. 2001) supplemented with 1:5 angiopoietin, stem cell factor (SCF) (10 ng/ml), fibroblast growth factor (10 ng/ml), thrombopoietin (20 ng/ml) and insulin-like growth factor (20 ng/ml) (PeproTech) (*39*). Cells were transduced using pMYs-*EVI1* (a gift of M. Kurokawa), pMYs-Δ*EVI1* (single mutation in PLDLS motif), or an empty vector control by RetroNectin transduction. In short, noncoated cell culture dishes (3.5 cm; Falcon, catalog no. 351008) were coated using 12 μg of RetroNectin (Takara, catalog no. T100B). Coated dishes then were pre-incubated with 1 ml of viral supernatant for 4 to 5 hours at 37°C. After removing the virus, 1 to 2 × 10^6^ HSC’s from an overnight culture were added to each dish. Cells were cultured overnight. This procedure was repeated for an additional 24 hours. After transduction, cells were plated in MethoCult (STEMCELL Technologies, catalog no. M3231) containing IL-3 (10 ng/ml), IL-6, granulocyte-macrophage colony-stimulating factor (GM-CSF), and SCF (all from PeproTech) at a concentration of 3 × 10^4^ cells/ml. Colonies were counted and replated every 7 days. Material harvested from serial replatings was used in expression analysis for *EVI1* by reverse transcription quantitative polymerase chain reaction. After four rounds of replating, *EVI1*-overexpressing cells were transferred to RPMI medium (Gibco, catalog no. 21875-034) containing IL-3 (10 ng/ml), IL-6, GM-CSF, and SCF (PeproTech) to establish a multiclonal cell line overexpressing *EVI1*. Data visualization and statistical testing were performed in GraphPad Prism (version 9.3.1).

### RNA sequencing

MUTZ3 cells were transduced with retroviral vectors encoding either p50MX-4× empty vector–Zeocin, p50MX-4× PLASS-Zeocin, or p50MX-4× PLDLS-Zeocin. RNA was isolated at 72 hours of Zeocin selection. RNA quality was determined, and 1 μg of RNA was used in library preparation using TruSeq RNA library preparation kit (Illumina, catalog no. no RS-122-2001). Library preparation was performed according to the manufacturer’s protocol. DNA was sequenced on a Illumina 2000-2500 platform, 75 cycles paired-end.

### RT-PCR of human and mouse EVI1

A minimum of 1 × 10^6^ cells were resuspended in 1 ml of TRIzol (Life Technologies, catalog no. 15596018), and RNA was isolated following the manufacturer’s protocol. RNA (1 μg) was used in RT using SuperScript II Reverse Transcriptase (Life Technologies, catalog no. 18064-014). Human or mouse *EVI1* PCR was carried out using specific primer sets (see table S1) using an ABI7500 real-time PCR cycler. Expression was normalized using mouse *Hprt* or human *PBGD*.

### WB analysis

Cells were lysed in Sarin buffer (20 mM tris-HCl, 138 mM NaCl, 10 mM EDTA, 50 mM NaF, 1% Triton, and 10% glycerol), which was supplemented with protease inhibitors SigmaFast and Na3VO4 and reducing agent dithiothreitol (DTT) (all chemicals purchased from Sigma- Aldrich). Protein content was measured by Pierce BCA protein Assay (Thermo Fisher Scientific, catalog no. 23227). Protein (40 μg) was loaded on a 4 to 12% Bis-Tris polyacrylamide gel (Thermo Fisher Scientific, catalog no. NP0321) and ran in a Mini Gel Tank (Thermo Fisher Scientific) in 1X Mops buffer (Thermo Fisher Scientific catalog no. NP001). Proteins were semi-dry blotted onto a 0.2 μM nitrocellulose membrane (Sigma-Aldrich, catalog no. GE10600001), and protein levels were detected by specific antibodies directed against Human EVI1 (Cell Signaling Technology, catalog no. 2265), CTBP1 (BD, catalog no. 612042), CTBP2 612044 (BD, 612044), V5 tag (Life Technologies, catalog no. R96025), FLAG tag (Sigma-Aldrich, catalog no. F3165), β-actin (Sigma-Aldrich, catalog no. no A5441), and hemagglutinin tag (Santa Cruz Biotechnology, catalog no. no Sc-805). Proteins were visualized using the Odyssey infrared imaging system (LI-COR Biosciences).

### Flow cytometry

Flow cytometric analysis of mouse bone marrow cells or MUTZ3 cells was done with specific antibody stainings using mouse CD11B-allophycocyanin (BD, catalog no. 553311) and mouse Gr- 1 FITC (BioLegend, catalog no. 108406). Cells were analyzed on a BD LSRII flow cytometer (BD Bioscience). Data were analyzed with FlowJo (dotplots and frequency tables). All other graphs were made in ggplot.

### ChIP sequencing

ChIP was performed in MUTZ3, MOLM1, and primary AML cells using specific antibodies against human EVI1 (Cell Signaling Technology, catalog no. 2593) and CTBP2 (BD, 612044). Cells were crosslinked for 45 min using 0.5 μM DSG (Thermo Fisher Scientific, catalog no. 20593). Cells were pelleted, resuspended in phosphate-buffered saline (PBS), and crosslinked for 10 min using 1% formaldehyde (500 ml; Sigma-Aldrich, catalog no. F8775). Formaldehyde crosslinking was quenched by adding 1.25 M glycin 5 min at room temperature. Cells were washed three times using cell lysis buffer containing 10 mM tris-HCl (pH 7.5), 10 mM NaCl, 3 mM MgCl, and 0.5% NP-40. After last wash, cells were resuspended in sonication lysis buffer with 0.8% SDS, 160 mM NaCl, 10 mM tris-HCl (pH 7.5), 10 mM NaCl, 3 mM MgCl, 1 mM CaCl_2_, and 4% NP-40. Lysates were sonicated on a Biorupter Pico (Diagenode). Chromatin was diluted four to five times using IP dilution buffer containing 1.1% Triton X-100, 0.01% SDS, 167 mM NaCl, 16.7 mM tris-HCl (pH 8.0), and 1.2 mM. A 2% sample was saved as an input control. Antibody (5 μg) was added, and chromatin was mixed overnight at 4°C. The following day, 30 μl of protein G beads (Thermo Fisher Scientific, catalog no. 1004D) was added to the samples and mixed for an additional 3 hours. Washing of the beads and DNA elution was performed following the standard ChIP protocol from Upstate. (All chemicals in buffers are purchased at Sigma-Aldrich). Libraries for sequencing were generated by using either the TruSeq ChIP (BIOO Scientific/PerkinElmer, catalog no. nova-5143-01) or MicroPlex Library v3 (Diagenode, catalog no. C05010001) preparation kit according to the manufacturer’s protocol. Samples were single-end sequenced [1 × 50 base pair (bp)] on the HiSeq 2500 platform (Illumina) or paired-end (2 × 100 bp) on the NovaSeq 6000 platform (Illumina).

### MAPPIT assay

HEK293T cells were seeded at 0.35 × 10^6^ cells per well in a six-well plate. The next day, cells were transfected with 1 μg of pXP2d2-rPAP1-luciferase (STAT3-responsive luciferase reporter), 1 μg of pCEL (the modified EPO receptor; bait) and 1 μg of pMG2 (the gp130 fusion needed to activate STAT3 at the modified receptor; prey) in 250 μl of DMEM-/-/- medium containing 9 μl of Lipofectamine 2000 (Invitrogen, 11668500). For each condition, a positive control (MG2-RNF) was also transfected to which data are normalized and which represents maximum reporter induction. DNA, media, and Lipofectamine are incubated for 5 min at room temperature and added dropwise to cells. After 48 hours of transfection, each well was trypsinized and divided over four wells in a 96-well plate in a total volume of 100 μl, and cells were incubated overnight with EPO (4 U/μl) to induce JAK/STAT signaling for 24 hours. Luciferase activity was measured with addition of 50 μl of Steady-Glo (Promega catalog no. E2510) and incubated for 20 min, and luminescence was measured with a Victor X4 (PerkinElmer). For each experiment, replicates were collapsed when baseline (−EPO) was subtracted, and data were normalized to % induction of the positive control (RNF) (−EPO was 0). Experiments from independent dates were combined, and significance was tested in a two-way analysis of variance (ANOVA) (or mixed model in the case of missing values). *P* values reported are adjusted for multiple testing. Data visualization and statistical testing were performed in GraphPad Prism (v 9.3.1).

### shRNA-mediated gene knock down

Lentiviral supernatants were generated as described earlier in this manuscript. SB1690CB cells were transduced with short hairpin RNA (shRNA) constructs targeting *EVI1*, *CTBP2*, or a control shRNA, which was not able to target any mouse or human genes (table S1 contains shRNA sequences). The procedure to introduce shRNA constructs was similar to that off overexpression of *EVI1* described above. After transduction, cells were placed on puromycin selection at 1 μg/ml. Knockdown of target genes was determined by performing WB analysis on days 2 and 8. Proliferation analysis was performed by counting cells every 2 days and splitting cells back to equal cells numbers across all conditions.

### Mass spectrometry

#### Nuclear protein extractions for MS

Nuclear extractions were performed as previously described (*40*). Briefly, cells were spun down (500*g* for 5 min at 4°C) and washed twice in cold PBS. Pellet volume was determined using a standard, and cells were resuspended in five volumes of cold buffer A [10 mM Hepes KOH (pH 7.9), 1.5 mM MgCl_2_, and 10 mM KCl], incubated for 10 min on ice, and centrifuged (400*g* for 5 min at 4°C). Cell volume was determined again, and two volumes of cold buffer A++ [Buffer A + cOmplete protease inhibitors (Roche, catalog no. 04693159001) and 0.15% NP-40]. Cell suspension was transferred to a precooled buffer A calibrated dounce homogenizer. Ten strokes (4×) with a type B pestle (tight) were perfomed, and the douncer was left to cool for 30 s after each 10th stroke. The suspension spun down for 15 min at 3900 rpm (large centrifuge), and the cytoplasmic extract (the supernatant) was not used in this study. The pellet was washed in in 2 to 3 ml of cold PBS by flicking the tube and spun down again at 3900 rpm for 5 min. The pellet size was determined with the standard, and two volumes of cold buffer C+++ [420 mM NacL, 20 mM Hepes KOH (pH 7.9), 20% (v/v) glycerol, 2 mM MgCl_2_, 0.2 mM EDTA, 0.1% NP-40, cOmplete protease inhibitors (see buffer A++), and 0.5 mM DTT] were added and vigorously resuspended (not vortexed). The lysate was rotated for 1 hour at 4°C and spun down (Table Top centrifuge, 14,000 rpm for 30 min at 4°C), and the supernatant (nuclear extracts) was collected, snap-frozen, and stored at −80°C until further use.

#### IP of EVI1 for MS and WB

Concentration of nuclear protein extracts was determined using the Bradford assay (Thermo Fisher Scientific, catalog no. 23238). All MS-IP experiments were done in triplicate, with cell lysates obtained on separate dates, and the IP was performed simultaneously. All steps were performed on ice and all centrifuge steps at 4°C. For each IP on nuclear lysate, 1 mg of nuclear lysate was adjusted to 500 μl in protein incubation buffer [PIB+++; 150 mM NaCl, 50 mM tris (pH 8.0), cOmplete protease inhibitor (Roche, catalog no. 04693159001), 0.25% NP-40, and 1 mM DTT)]. For WB-IP on total cell lysate, the IP and all washing steps were performed in the protein extraction Sarin buffer (see WB). ProtG magnetic Dynabeads (30 μl; Thermo Fisher Scientific, catalog no. 1003D) were washed three times in 1 ml of PIB+++ or Sarin buffer. Lysates were precleared by mixing head over head for 2 hours at 4°C. For input samples for WB, 80 μg of protein lysate was heated at 95°C for 5 min in 40 μl of 1x Protein loading buffer (sample buffer for SDS–polyacrylamide gel electrophoresis gels). For the IP, 50 μl of ProtG Dynabeads (see above) were washed in 3 × 1 ml of PIB+++ or Sarin and added to the precleared lysates. Primary antibody (5 μg) was added [IgG control: Cell Signaling Rabbit (DA1E) mAb IgG XP Isotype Control #3900 Lot5; EVI1: Cell Signaling C50E12 Anti-EVI1 Rabbit IgG #2593S Lot3, CTP2: BD, 612044], and samples were mixed head over head overnight at 4°C. Samples were washed five times in 1 ml of cold PBS + 0.1% NP-40 or Sarin buffer and processed for MS. For WB samples, proteins were eluted from the beads with 30 μl of 1.5x Protein loading buffer and heated 10 min to 95°C while shaking and loaded onto a WB gel (see the “WB” section).

#### MS analysis

Nanoflow LC-MS/MS was performed on an EASY-nLC system (Thermo Fisher Scientific) coupled to a Orbitrap Fusion Lumos Tribrid mass spectrometer or an Orbitrap Eclipse Tribrid mass spectrometer (both Thermo Fisher Scientific) operating in positive mode and equipped with a nanospray source. Peptide mixtures were trapped on a ReproSil C18 reversed phase column (Dr Maisch GmbH; column dimensions, 1.5 cm by 100 μm, packed in-house) at a flow rate of 8 μl/min. Peptide separation was performed on ReproSil C18 reversed phase column (Dr Maisch GmbH; column dimensions, 15 cm by 50 μm, packed in- house) using a linear gradient from 0 to 80% B (A = 0.1% formic acid (FA); B = 80% (v/v) acetonitrile (AcN), 0.1% FA) in 120 min and at a constant flow rate of 250 nl/min. The column eluent was directly sprayed into the ESI source of the mass spectrometer.

For data-dependent acquisition (DDA), all mass spectra were acquired in profile mode. The resolution in MS1 mode was set to 120,000 (automatic gain control target: 4 × 10^5^), and the mass/charge ratio (*m*/*z*) ranges from 350 to 1400. Fragmentation of precursors was performed in 2-s cycle time data-dependent mode by high-energy collisional dissociation with a precursor window of 1.6 *m*/*z* and a normalized collision energy of 30.0; MS2 spectra were recorded in the orbitrap at 30,000 resolution. Singly charged precursors were excluded from fragmentation, and the dynamic exclusion was set to 60 s.

#### Quantification

DDA raw data files were analyzed using the MaxQuant software suite [version 2.2.0.0 (*41*)] for identification and relative quantification of proteins. “Match between runs” was disabled, and a false discovery rate (FDR) of 0.01 for proteins and peptides and a minimum peptide length of six amino acids were required. The Andromeda search engine was used to search the MS/MS spectra against the *Homo sapiens* Uniprot database (version May 2022) concatenated with the reversed versions of all sequences and a contaminant database listing typical background proteins. A maximum of two missed cleavages were allowed. MS/MS spectra were analyzed using MaxQuant’s default settings for Orbitrap and ion trap spectra. The maximum precursor ion charge state used for searching was 7, and the enzyme specificity was set to trypsin. Further modifications were cysteine carbamidomethylation (fixed) and methionine oxidation. The minimum number of peptides for positive protein identification was set to 2. The minimum number of razor and unique peptides was set to 0. Only unique and razor nonmodified, methionine oxidized and protein N-terminal acetylated peptides were used for protein quantitation. The minimal score for modified peptides was set to 40 (default value).

DIA raw data files were analyzed with the Spectronaut Pulsar X software package (Biognosys, version 17.0.221202) using directDIA for DIA analysis including MaxLFQ as the LFQ method and Spectronaut IDPicker algorithm for protein inference. The *q* value cutoff at precursor and protein level was set to 0.01. All imputation of missing values was disabled.

### Data analysis

#### Statistical analysis

Statistical analysis is specified in the corresponding method section of the analysis type concerned. In general, for colony assays or plate-based data such as MAPPIT, GraphPad Prism is used with two-way ANOVA (with appropriate multiple testing corrections). For other data types, specialized statistical software is used (DEseq2 for RNA-seq, DiffBind/Genomation for ChIP-seq data, Perseus/DEP for mass-spectrometry data).

#### Data visualization

Analysis have been conducted using R version 4.2.2 and visualized with ggplot2 unless otherwise specified.

#### Differential enrichment analysis MS

Perseus (version 2.0.3.1) was used for differential enrichment analysis. Log2 LFQ intensities from MaxQuant were loaded into Perseus. Common contaminants were excluded, and rows were filtered that contained values in at least three samples within a group. Missing values were replaced by a value below the lowest detected protein. Differential enrichment (DE) analysis was performed with the following parameters: program, EdgeR; test method, likelihood ratio test; normalization method EdgeR, TMM). Data for volcano plots was generated with a *t* test on the normalized data (parameters: side, both; number of randomizations, 250; preserve grouping in randomizations, none; FDR threshold, 0.05). The data were exported from Perseus and visualized in R using ggplot2. Biogrid database was used to identify previously identified proteins (version 4.4) (*17*). Complex enrichment was performed with EnrichR with the CORUM database (*20*). For the network analysis, a graph of proteins from the Perseus analysis (nodes) with connections (edges) representing their co-occurrence in the CORUM complexes was constructed using the Fruchterman-Reingold layout algorithm. The edges were weighted on the basis of the cosine distance between proteins in terms of how frequently they co-occur in the same complex. Clusters or communities of proteins with shared complexes were detected using the Louvain algorithm with default resolution. These analyses were conducted with the tidygraph and igraph R packages and visualized with ggraph. For the MS experiment in NFS78 (fig. S1D), data from three independent experiments were pooled. All control samples (NFS78 untransduced, Bir-A only) were compared to experimental samples (EVI1-Biotag + BirA expressing clones). Before importing them into Perseus, Mascot scores of each protein isoform were summarized at gene level (mean). The SD as % of the mean per resulting gene was inspected, showing that the variability for the vast majority of detected proteins was very small. Resulting tables were merged on the basis of the gene symbol, mascot scores were log_2_- transformed, and proteins were filtered on the basis of being detected in all experimental samples in all experiments. Missing values were replaced by the minimum detected log_2_ value of −1. Correlation between samples from the same experimental group was increased with this method, and missing values were greatly reduced. Data were imported into Perseus, analyzed as described above, and visualized with ggplot2.

For comparing the EVI1 interactome of 4× PLASS- versus 4× PLDLS-expressing MUTZ3 cells, the doxycycline-inducible system was used. Cells were induced with doxycycline (1 μg/ml) for 72 hours before harvest. To compare differential abundances within the EVI1 IP, DIA quantified data were used in the Differential Enrichment for Proteomics package (DEP) (*42*). Missing values were imputed from a normal probability distribution centered around a minimal value (minProb in DEP).

#### Data analysis ChIP-seq

ChIP-seq reads were aligned to the human reference genome build hg19 with bowtie (v1.1.1) (*43*). Peaks were called with MACS2 with default parameters and a matched input file (same ChIP protocol on the same cells) for transcription factors. In addition, the –broad argument was used for peak calling on histone marks (*44*). For heatmaps, the R package Genomation (to compare tracks at baseline without replicates) (version 1.30.0) (*45*) or DiffBind (to compare tracks in different conditions with replicates) (*46*) (version 3.8.4) was used. For heatmaps made in DiffBind, differentially bound sites were calculated using default normalization and heatmap options. The consensus peaksets derived by DiffBind were used as input for the heatmaps, and replicates were collapsed into a single heatmap lane (with dba.plotProfile). For heatmaps made in Genomation, peaks called by MACS2 were sorted on either EVI1 enrichment or CTBP2 enrichment. These references peaks were widened symmetrically to 1000 bp on each side and used as windows to count signal in the BAM files of all tracks shown. The heatmaps are winsorized (“overexposed”) on (0,99), meaning all signal above the 99th percentile of peaks are the same color. H3K27Ac tracks were winsorized on the 90th percentile. To quantify correlation in the heatmaps, DiffBind was used to count and normalize reads in all tracks within either EVI1 or CTBP2 peaks that were widened to 1000 bp on each side. Reads were normalized with default DiffBind parameters, and correlation was calculated with linear regression on log_10_-transformed data and a pseudocount of 1. Correlation was visualized in ggplot2 with geom_hex. Individual loci were visualized with Gviz (version 1.42.1) (*47*). To annotate peaks to genes, deoxyribonuclease (DNase) hypersensitive sites significantly correlated with gene expression across diverse tissues were used (*48*). If peaks did not overlap directly with a DNase site in this dataset, they were annotated to the nearest protein-coding gene with ChIPpeakAnno (*49*) (version 3.32.0). To calculate fold changes for CTBP2 and EVI1 binding, DiffBind was used (same as above).

#### Data analysis RNA-seq

RNA-seq reads were quantified from fastq files directly using Salmon (v0.13.1) using hg38 RefGene database as an index. Transcript-level counts were aggregated to genes with tximport (v1.26.1), and differential gene expression analysis was performed with DEseq2 (v1.38.3). A significance cutoff of log_2_FC > 1 and adjusted *P* value < 0.05 were used to determine the set of DE genes.

#### AlphaFold

AlphaFold (v2.2.0, database updated to 08-01-2023) was run locally with 5 × 5 iterations and default parameters. Using Python, for each predicted relaxed multimer model, interaction residues were quantified with “interfaces,” “interfaces areaCutOff 0,” or “hbonds” in ChimeraX. The information for selecting residues was written to the log and extracted per structure. The resulting log files were important into R and combined into a single file with for each structure. The number of residues in each chain predicted to be interacting with each other was quantified, and,if any, their identity was extracted. Data were visualized with ggplot2. 3D visualizations were done for the top ranked relaxed multimer models in ChimeraX.

#### DEPMAP analysis

Public data from the 23Q2 release of the DEPMAP consortium (https://depmap.org/portal/). Gene effect for selected genes (CRISPRGeneEffect), as well as expression, was plotted. Blood/bone marrow (BM) cell lines were identified with the pattern “Leukemia|Myeloid|H(e|ae)matopoetic|Blood|Marrow|AML” in any of the columns CCLEname, DepmapModelType, OncotreeCode, OncotreeSubtype, OncotreePrimaryDisease, OncotreeLineage.

#### Protein sequence alignment

To find PLDLS-containing proteins, BLAST was used to query “PLDLS” against the human RefSeq protein database. Per protein, the sequence of the PLDLS site −15/+15 amino acids was extracted and aligned using the msa package in R. Distances for the clustering tree were computed with dist.alignment (seqinr) using parameter “similarity,” which calculates the sample-to-sample distances based on amino acid characteristics (Fitch matrix) (*50*). The tree itself was computed with hclust and plotted with plot.phylo. Protein alignments and all other plots [subcellular localization (retrieved with bioMart), CTBP1/2 interactors from BioGrid (v4.4.222), protein expression from DIA-quantified input MS samples] were plotted with ggplot. For predictions with AlphaFold, the extracted sequences were written to .fasta files together with the full CTBP2 sequence. This was performed and analyzed as above.

### Xenotransplant models

#### Transplantation of SB1690CB cells into in NSG mice

Lentiviral supernatants of vectors expressing 4× PLDLS, 4× PLASS, or empty controls, labeled with Emerald-GFP or mCherry, were produced as described above. SB1690CB cells were transduced at multiplicity of infection (MOI) = 2 48 hours before injection into mice. The proportion of labeled cells was ascertained by flow cytometry before cells suspensions being combined to produce mixtures containing approximately 1:1 Emerald-GFP:mCherry–labeled cells in three groups: Emerald-empty vector versus mCherry-empty vector, 4× PLASS Emerald versus 4× PLDLS mCherry, or 4× PLASS mCherry versus 4× PLDLS Emerald. For competitive engraftment, cell mixtures were administered into sublethally irradiated 8- to 12-week-old female NSG mice (NOD/NSG/IL-2Rgamma chain null) via intrabone injection of ~1 × 10^6^ filtered cells and suspended in 40 μl of sterile PBS (Gibco, 10010056) containing 0.5% heat-inactivated fetal bovine serum (FBS) (Gibco, 16140071). Mice were euthanized after 10 weeks, and suspensions of bone marrow cells were prepared. After filtering, cells were washed twice and resuspended in PBS containing 2% FBS before staining in the presence of Fc block (Miltenyi, 130-059-901) with anti-human CD45 Alexa Fluor 700 (clone HI30, BioLegend, catalog no. 304024) and anti-mouse CD45.1 phycoerythrin-Cy7 (clone A20, BioLegend, catalog no. 110730) antibodies and Hoechst 33258 (Thermo Fisher Scientific, catalog no. H3569). Cells were analyzed using a BD FACSAria III Cell Sorter (BD Bioscience). The ratio of mCherry to Emerald-GFP–expressing cells was determined as a proportion of human CD45-positive cells, following gating to exclude dead cells, doublets, and murine CD45.1^+^ cells. Data were acquired using BD FACSDiva software v 8.0.1 (BD Bioscience), and tables of cell populations gated was exported from FACSDiva and prepared for analysis using the pandas data analysis library for Python. Frequency tables were visualized with ggplot. Xenograft experiments were performed with ethical approval under the Enver lab REC reference: 12/NW/0909. Care of animals, administration of cells, and animal sacrifice were performed in accordance with U.K. Home Office personal and project license requirements.

#### Transplantation of MUTZ3 into human bone marrow–like scaffold mice

Human bone marrow–like scaffolds were prepared as described previously (*27*, *51*) with modifications in scaffold shape, ceramic material, and the addition of human vasculature. Briefly, tube-shaped β-tricalcium phosphate ceramic scaffolds were loaded with 1 × 10^6^ expanded healthy donor bone marrow mesenchymal stromal cells (MSCs) and incubated for 7 days in osteogenic medium. For the generation of human vessels, mixtures of human 5 × 10^5^ MSC and 5 × 10^5^ human cord blood–derived endothelial colony forming cells (ratio 1:1) were grown in matrigel (BD Biosciences) and injected into the cavity of the tubes and stored at 37°C for 30 min before subcutaneous implantation into RAG2^−/-^γC^−/−^ mice. Eight weeks after implantation mice were irradiated, and 1 day later 10 × 10^6^ luciferase-marked MUTZ3 cells, transduced with a retroviral 4× PLASS-Puro or 4× PLDLS- Puro vector as described above, were injected directly into the tubes. Every 6 to 8 days, mice were anesthesized with isoflurance and intraperitoneally injected with 100 μl of 7.5 mM beetle luciferin (Promega). Bioluminescent imaging was performed during tumor growth using an intensified charge-coupled device (CCD) camera (ICCD) operating in a photon counting mode (Photon Imager, Biospace Lab, France) controlled by Photo Vision software and analyzed with M3Vision software (Photon Imager, Biospace Laboratory) (*52*). Outgrowth data were normalized to day 0 and visualized with ggplot (geom_sina). All animal studies were approved by our Animal Ethical Committee [VUMc (now AUMC), Amsterdam, the Netherlands].

## Supplementary Materials

### This PDF file includes:

Figs. S1 to S7 Legend for table S1 Uncropped Western blots for panel S1A
