# Supplementary material for: Oncogene EVI1 drives acute myeloid leukemia via a targetable interaction with CTBP2
Source: Sci Adv. 2024 May 15;10(20):eadk9076. doi: 10.1126/sciadv.adk9076 (PMC11095456; doi:10.1126/sciadv.adk9076)

Science Advances
AAAS

Supplementary Materials for

Oncogene *EVI1* drives acute myeloid leukemia via a targetable interaction with CTBP2

Dorien Pastoors *et al.*

Corresponding author: Ruud Delwel, h.delwel@erasmusmc.nl

Sci. Adv. **10**, eadk9076 (2024)
DOI: 10.1126/sciadv.adk9076

The PDF file includes:

Figs. S1 to S7
Legend for table S1
Uncropped Western blots for panel S1A

Other Supplementary Material for this manuscript includes the following:

Table S1

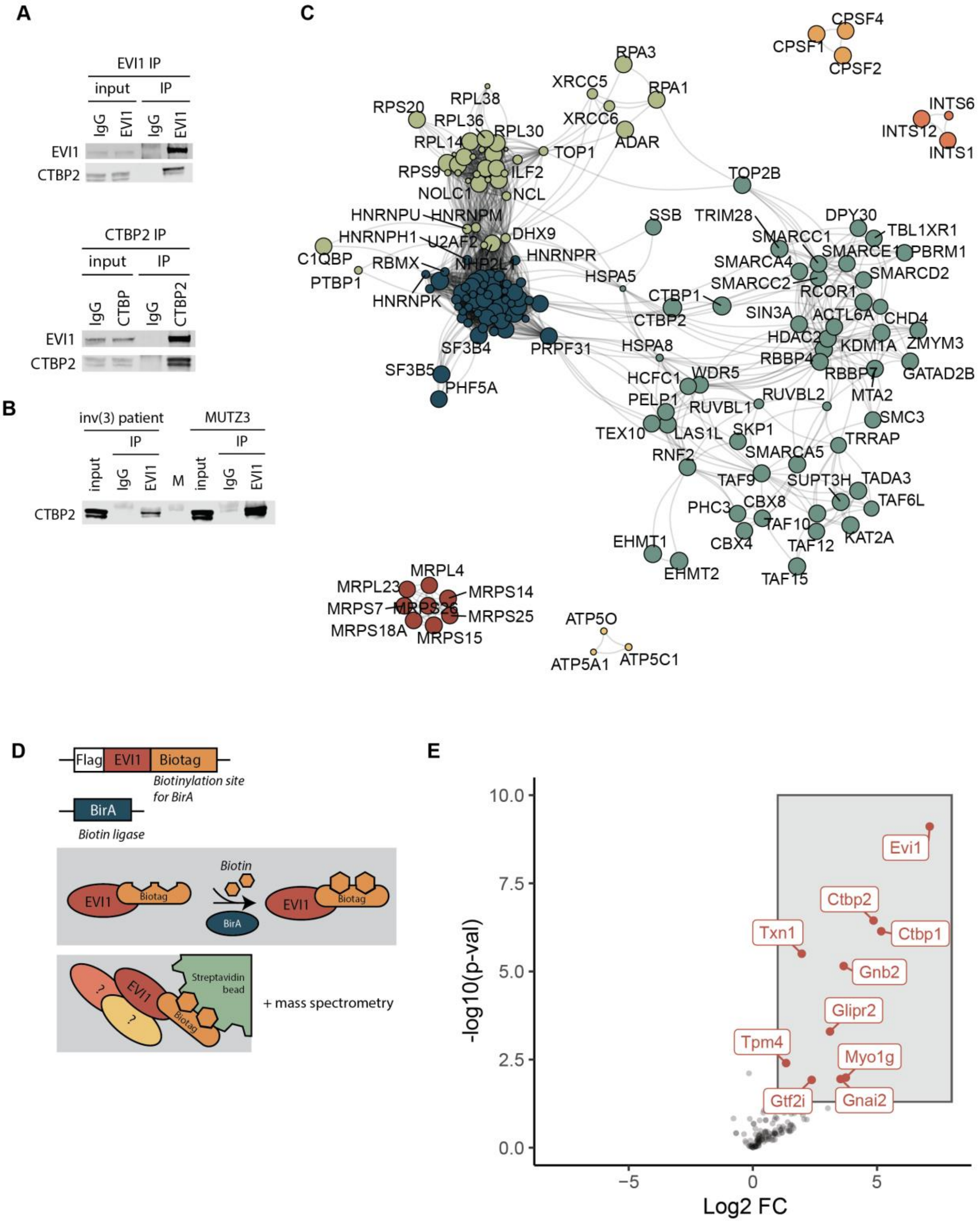
A
EVI1 IP
input
IP
IgG
EVI1
IgG
EVI1
EVI1
CTBP2
CTBP2 IP
input
IP
IgG
CTBP
IgG
CTBP2
EVI1
CTBP2
B
inv(3) patient
MUTZ3
IP
IP
input
IgG
EVI1
M
input
IgG
EVI1
CTBP2
C
CPSF4
CPSF1
CPSF2
INTS6
INTS12
INTS1
RPA3
RPA1
XRCC5
XRCC6
ADAR
RPL38
RPS20
RPL36
RPL30
RPL14
TOP1
RPS9
ILF2
NOLC1
NCL
HNRNPU
HNRNPM
HNRNPH1
U2AF2
DHX9
C1QBP
RBMX
NHP2L1
HNRNPR
PTBP1
HNRNPK
SF3B4
PRPF31
SF3B5
PHF5A
TOP2B
SSB
TRIM28
DPY30
TBL1XR1
SMARCC1
SMARCE1
PBRM1
SMARCA4
SMARCD2
HSPA5
SMARCC2
RCOR1
CTBP1
CTBP2
SIN3A
ACTL6A
CHD4
HDAC2
KDM1A
ZMYM3
HSPA8
RBBP4
RBBP7
GATAD2B
HCFC1
WDR5
MTA2
PELP1
RUVBL1
RUVBL2
SMC3
TEX10
LAS1L
SKP1
TRRAP
RNF2
SMARCA5
TAF9
SUPT3H
TADA3
TAF6L
PHC3
CBX8
TAF10
CBX4
TAF12
KAT2A
EHMT1
EHMT2
TAF15
MRPL4
MRPL23
MRPS14
MRPS7
MRPS26
MRPS25
MRPS18A
MRPS15
ATP5O
ATP5A1
ATP5C1
D
Flag
EVI1
Biotag
Biotinylation site for BirA
BirA
Biotin ligase
Biotin
EVI1
Biotag
BirA
EVI1
Biotag
?
EVI1
?
Biotag
Streptavidin bead
+ mass spectrometry
E
-log10(p-val)
10.0
7.5
5.0
2.5
0.0
Log2 FC
−5
0
5
Evi1
Ctbp2
Txn1
Ctbp1
Gnb2
Glipr2
Tpm4
Myo1g
Gtf2i
Gnai2

Figure S1: Supplement to figure 1 (Pt1)

Fig S1A. EVI1 IP followed by CTBP2 Western blot (top panel) or vice versa (bottom panel) in MUTZ3 whole-cell lysates.

Fig S1B. EVI1 IP followed by CTBP2 Western blot in inv(3) patient and MUTZ3 whole-cell lysates

Fig S1C. Full version of Fig 1B, including all the protein labels and groups of proteins removed because they are likely contaminants.

Fig S1D. Outline of EVI1 pulldown experiments with EVI1-Biotag and BirA overexpression in NFS78

Fig S1E. MS enrichment of proteins in streptavidin IP of BirA+ EVI1-Biotag versus BirA alone in murine cell line NFS78. (Significance cut-off: log2FC > 1 and p-value < 0.05 (n = 4 for each group))

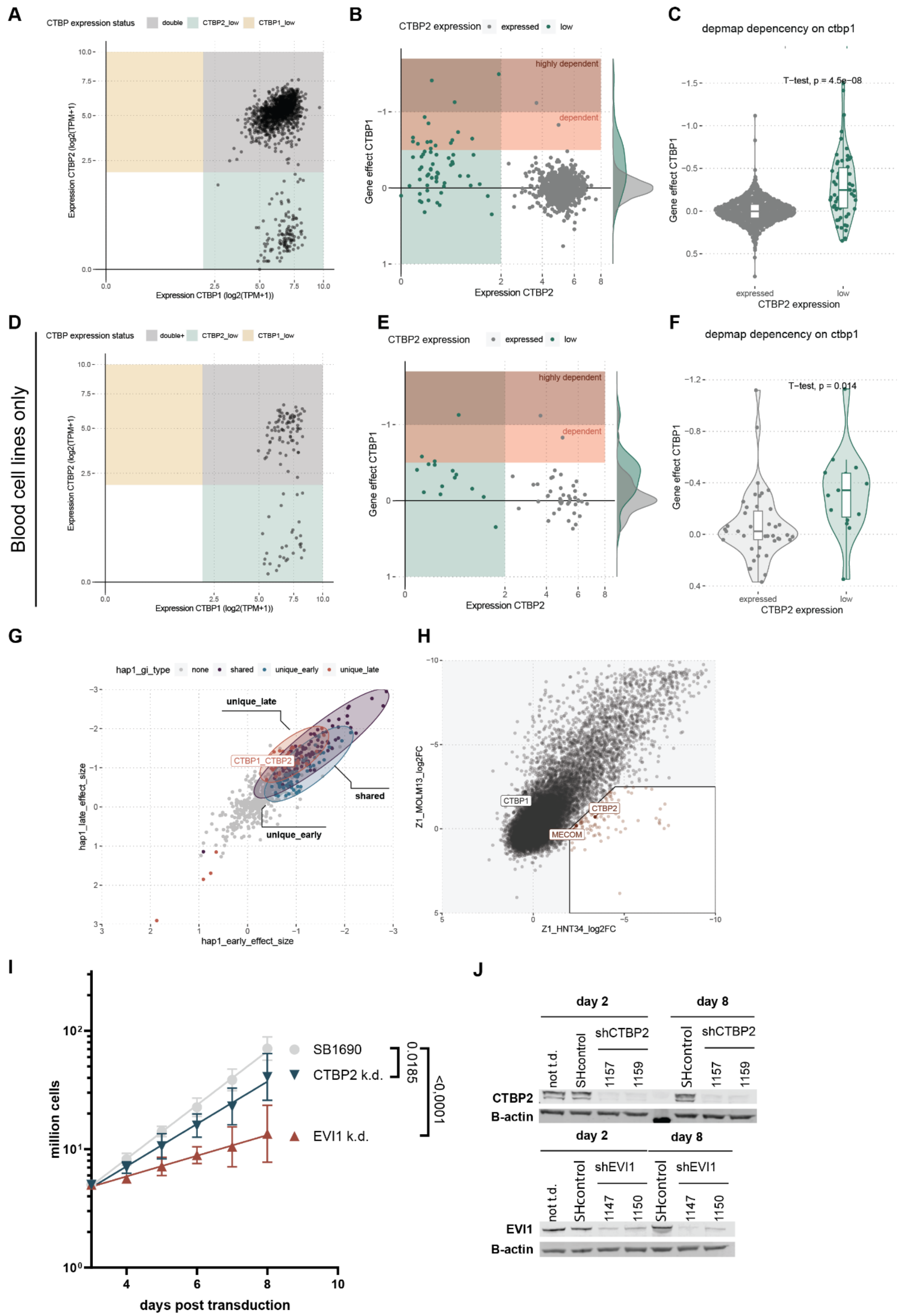

A
CTBP expression status double CTBP2_low CTBP1_low
Expression CTBP2 (log2(TPM+1))
Expression CTBP1 (log2(TPM+1))
B
CTBP2 expression expressed low
highly dependent
dependent
Gene effect CTBP1
Expression CTBP2
C
depmap depencency on ctbp1
T−test, p = 4.5e−08
Gene effect CTBP1
expressed
low
CTBP2 expression
D
Blood cell lines only
CTBP expression status double+ CTBP2_low CTBP1_low
E
CTBP2 expression expressed low
F
depmap depencency on ctbp1
T−test, p = 0.014
G
hap1_gi_type none shared unique_early unique_late
unique_late
CTBP1_CTBP2
shared
unique_early
hap1_late_effect_size
hap1_early_effect_size
H
CTBP1
CTBP2
MECOM
Z1_MOLM13_log2FC
Z1_HNT34_log2FC
I
million cells
days post transduction
SB1690
CTBP2 k.d.
EVI1 k.d.
0.0185
<0.0001
J
day 2
day 8
not t.d.
SHcontrol
shCTBP2
1157
1159
CTBP2
B-actin
shEVI1
1147
1150
EVI1

Figure S2. Supplement to Fig.1 (Pt2)

Fig S2A. Expression of CTBP1 and CTBP2 on cancer cell lines from DEPMAP. Quandrants indicate single-positive and double-positive cells.

Fig S2B. Expression of CTBP2 versus dependency on CTBP1. Quadrants indicate low expression of CTBP2 (teal, X-axis) and dependency on CTBP1 (shades of red, y-axis).

Fig S2C. Dependency on CTBP1 in cells that express CTBP2 (grey) and cells that express little CTBP2 (teal). A t-test is performed to assess significance between the groups.

Fig S2D. Fig. S2A, but filtered for blood cell lines only

Fig S2E. Fig. S2B, but filtered for blood cell lines only

Fig S2F. Fig. S3B, but filtered for blood cell lines only

Fig S2G. Visualisation of gene effect of paralog knock-outs versus single-gene knockouts at early and late timepoints in paralog screen in HAP1 cells (*39*) . The CTBP1_CTBP2 pair is labelled

Fig S2H. Visualisation of specific dependencies of EVI1+ HNT34 cells and EVI1- MOLM13 cells. The axes depict depletion of genes from HNT34 versus MOLM13 in a viability screen (*40*). CTBP1, CTBP2 and MECOM are labelled

Fig S2I. Growth curve in SB1690CB with two independent shRNAs per gene for CTBP2 and MECOM. To determine significance, an exponential growth model (Y= logY0 + k*X)) is fit and best-of-fit value for growth rate k is compared between groups with an extra sum-of-squares F-test. The control cells are untransduced and shcontrol-transduced cells (n = 2 per group; Mean + SD plotted).

Fig S2J. Western blot of knock-down efficiency of SB1690CB growth curve depicted in panel S2I.

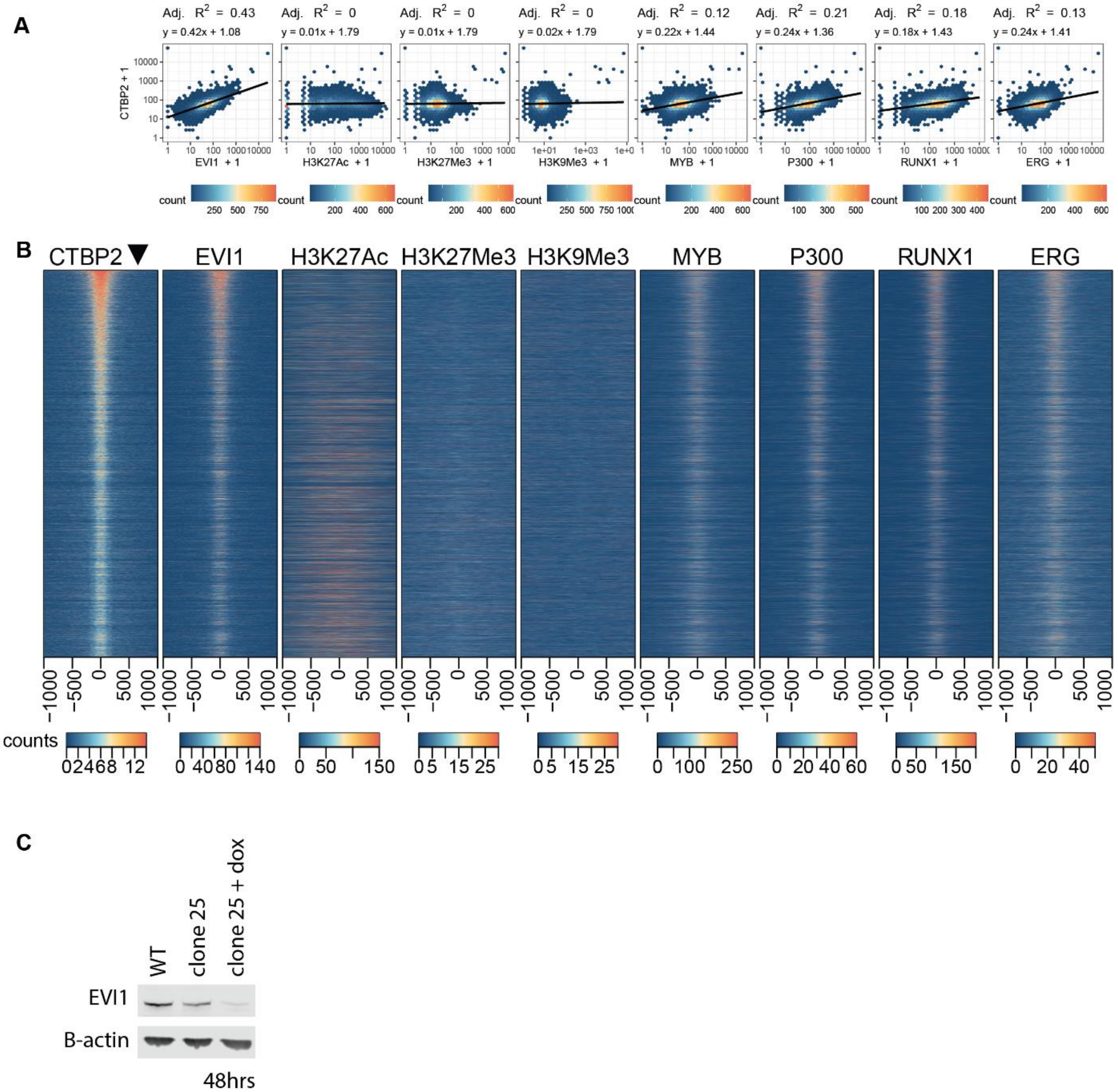

A
Adj. R^2 = 0.43
y = 0.42x + 1.08
Adj. R^2 = 0
y = 0.01x + 1.79
Adj. R^2 = 0
y = 0.01x + 1.79
Adj. R^2 = 0
y = 0.02x + 1.79
Adj. R^2 = 0.12
y = 0.22x + 1.44
Adj. R^2 = 0.21
y = 0.24x + 1.36
Adj. R^2 = 0.18
y = 0.18x + 1.43
Adj. R^2 = 0.13
y = 0.24x + 1.41
CTBP2 + 1
EVI1 + 1
H3K27Ac + 1
H3K27Me3 + 1
H3K9Me3 + 1
MYB + 1
P300 + 1
RUNX1 + 1
ERG + 1
count
B
CTBP2
EVI1
H3K27Ac
H3K27Me3
H3K9Me3
MYB
P300
RUNX1
ERG
counts
C
WT
clone 25
clone 25 + dox
EVI1
B-actin
48hrs

Figure S3. Supplement to Fig.1 (Pt3)

Fig. S3A. Quantification of the heatmap in panel Fig. S3B. For each track, the normalised CTBP2 signal is plotted versus the normalised reads in the indicated ChIP-seq-tracks, for all CTBP2 peaks with a window of ±1000bp. Correlation coefficients and linear regression equations are shown for log10-transformed data with a pseudo count of 1.

Fig. S3B. Heatmap ranked on ChIP-seq CTBP2 signal (leftmost panel) showing signal intensity of indicated ChIP-seq tracks within CTBP2 peaks (16836 peaks). Signal is capped at 90th percentile (H3K27Ac) or 99th percentile (all other tracks).

Fig. S3C. Western blot of inducible *EVI1* knock-down in a clone derived from MOLM1 performed in the same experiment as the ChIP-seq in Fig. 1E (48 hrs of doxycycline)

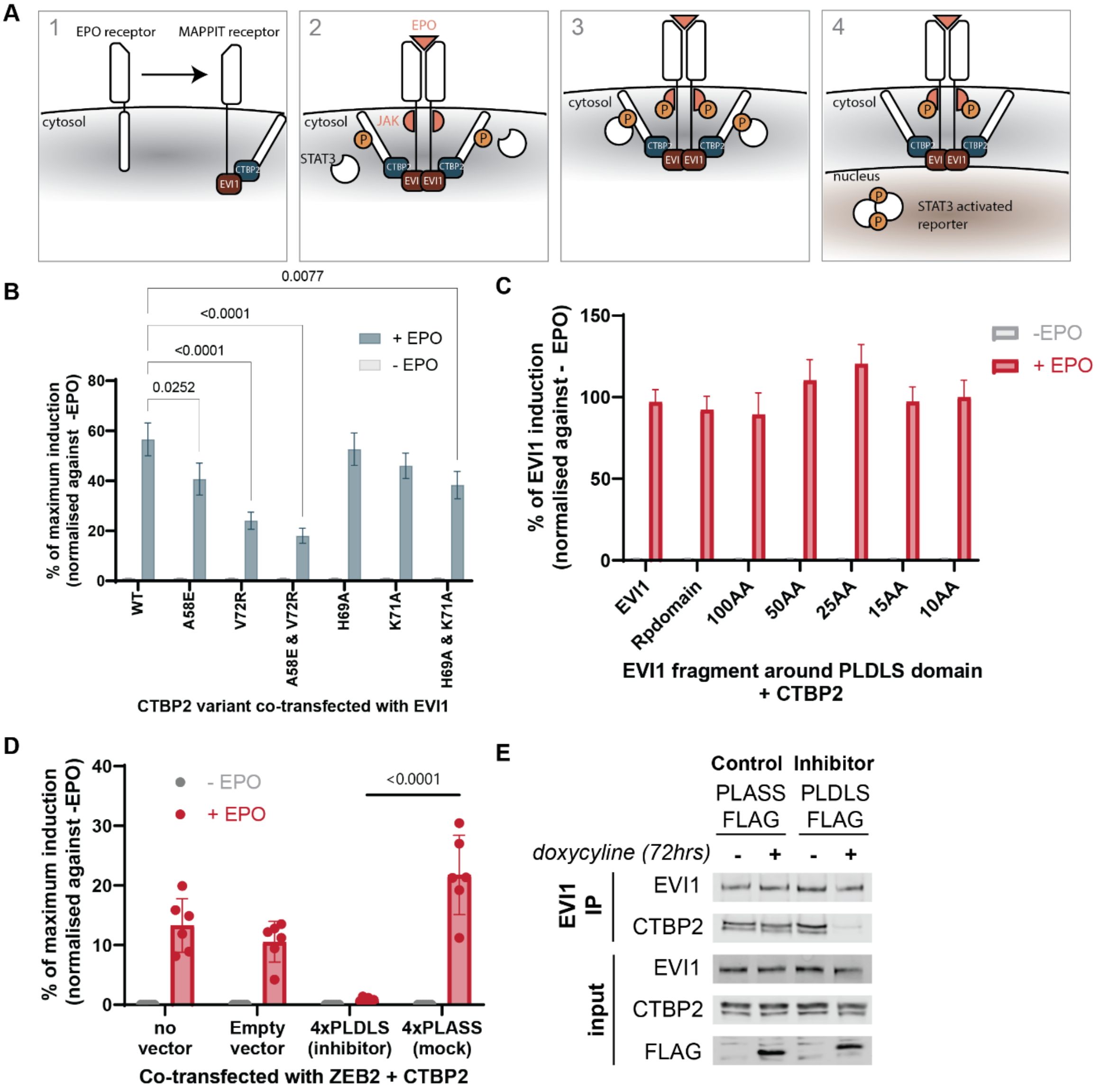
A
1
EPO receptor
MAPPIT receptor
cytosol
2
EPO
JAK
STAT3
3
4
nucleus
STAT3 activated reporter
B
% of maximum induction (normalised against -EPO)
+ EPO
- EPO
0.0077
<0.0001
<0.0001
0.0252
WT
A58E
V72R
A58E & V72R
H69A
K71A
H69A & K71A
CTBP2 variant co-transfected with EVI1
C
% of EVI1 induction (normalised against - EPO)
-EPO
+ EPO
EVI1
Rpdomain
100AA
50AA
25AA
15AA
10AA
EVI1 fragment around PLDLS domain + CTBP2
D
% of maximum induction (normalised against -EPO)
- EPO
+ EPO
<0.0001
no vector
Empty vector
4xPLDLS (inhibitor)
4xPLASS (mock)
Co-transfected with ZEB2 + CTBP2
E
Control
Inhibitor
PLASS FLAG
PLDLS FLAG
doxycyline (72hrs)
EVI1 IP
input
EVI1
CTBP2
FLAG

Figure S4: Supplement to figure 2-3

Fig. S4A. Schematic outline of the MAPPIT assay to measure protein-protein interaction (here for EVI1 and CTBP2). The MAPPIT receptor is based on the EPO receptor, which harbors an intracellular domain that can be phosphorylated by JAK in normal JAK-STAT signalling (1). In the modified MAPPIT receptor, the intracellular domain fused to EVI1 and the Tyr phosphorylation sites are mutated. Gp130 is fused to CTBP2 (2). Because EVI1-CTBP2 can interact with each other, CTBP2 is recruited to the EPO-EVI1 receptor. In this way, when signal is transmitted by ligand binding (2), JAK will phosphorylate tyrosines on CTBP2-gp130 to subsequently activate STAT3 (3). Phosphorylated STAT3 is transported into the nucleus where it will activate a STAT3 responsive luciferase reporter (4).

Fig. S4B. MAPPIT assay in HEK293T cells to measure EVI1-CTBP2 interaction, with additional mutations in residues in CTBP2 based on directly interacting residues in AlphaFold predictions. Reporter induction is normalised to –EPO (not induced) and maximum induction with RNF as positive control. Statistical significance is determined with a two-way ANOVA (significance shown if $P<0.05$; 1 independent experiments quadruplicate; mean + SEM is shown).

Fig. S4C. MAPPIT assay on HEK293T cells of EVI1 and CTBP2, with amino acid (AA) segments of varying sizes around the PLDLS site (100AA is ±100AA) (n=1 in triplicate).

Fig. S4D. MAPPIT assay on HEK293T cells (n=6, in triplicate) of ZEB2 and CTBP2, with inhibitor of EVI1 and CTBP interaction. Reporter induction normalised to –EPO (not induced) and maximum induction with RNF positive control. Significance with two-way ANOVA (comparison PLDLS vs PLASS + EPO shown).

Fig. S4E. IP EVI1-WB CTBP1/2 on MUTZ3 cells, stained for EVI1 and CTBP2 with 4x PLASS-FLAG or 4x PLDLS-FLAG dox-inducible overexpression construct.

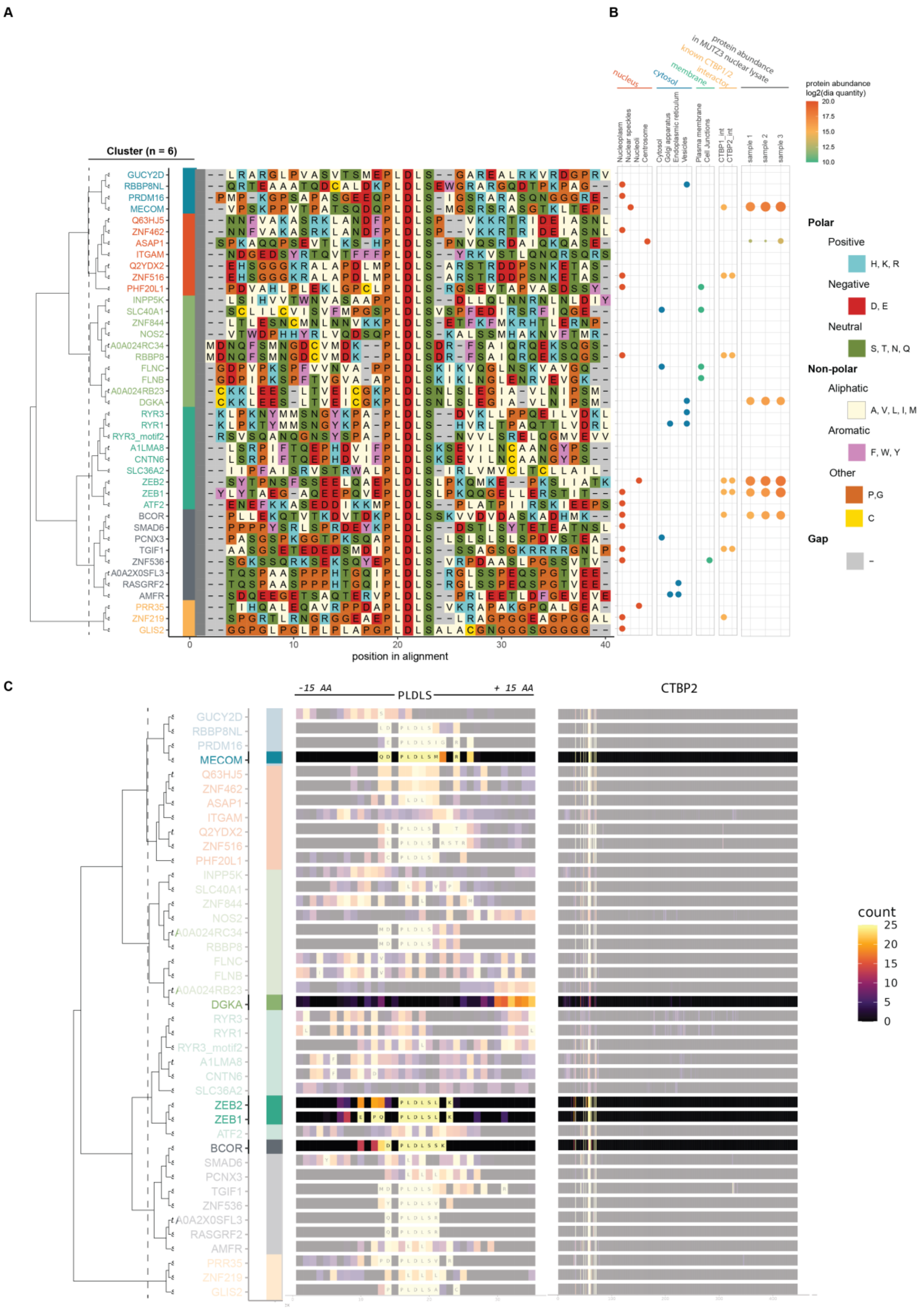

A
B
C
Cluster (n = 6)
GUCY2D
RBBP8NL
PRDM16
MECOM
Q63HJ5
ZNF462
ASAP1
ITGAM
Q2YDX2
ZNF516
PHF20L1
INPP5K
SLC40A1
ZNF844
NOS2
A0A024RC34
RBBP8
FLNC
FLNB
A0A024RB23
DGKA
RYR3
RYR1
RYR3_motif2
A1LMA8
CNTN6
SLC36A2
ZEB2
ZEB1
ATF2
BCOR
SMAD6
PCNX3
TGIF1
ZNF536
A0A2X0SFL3
RASGRF2
AMFR
PRR35
ZNF219
GLIS2
position in alignment
nucleus
cytosol
membrane
known CTBP1/2 interactor
protein abundance in MUTZ3 nuclear lysate
Nucleoplasm
Nuclear speckles
Nucleoli
Centrosome
Cytosol
Golgi apparatus
Endoplasmic reticulum
Vesicles
Plasma membrane
Cell Junctions
CTBP1_int
CTBP2_int
sample 1
sample 2
sample 3
protein abundance log2(dia quantity)
Polar
Positive
H, K, R
Negative
D, E
Neutral
S, T, N, Q
Non-polar
Aliphatic
A, V, L, I, M
Aromatic
F, W, Y
Other
P,G
C
Gap
-15 AA
PLDLS
+ 15 AA
CTBP2
count

Figure S5. Supplement to Fig. 3

Fig S5A. Protein alignment of all PLDLS sites +- 15 AA from the human proteome. Distance clustering is based on amino acid similarity (Fitch matrix).

Fig S5B. Subcellular localisation of PLDLS containing proteins, whether they have been identified previously as CTBP1/2 interactors (Based on BioGrid) and protein expression from DIA-quantified mass spectrometry nuclear lysate input samples.

Fig S5C. ChimeraX interaction on AlphaFold predicted heterodimer models of CTBP2 with the PLDLS site from the indicated protein. The count refers to the number of predicted models where the interaction with this residue was found.

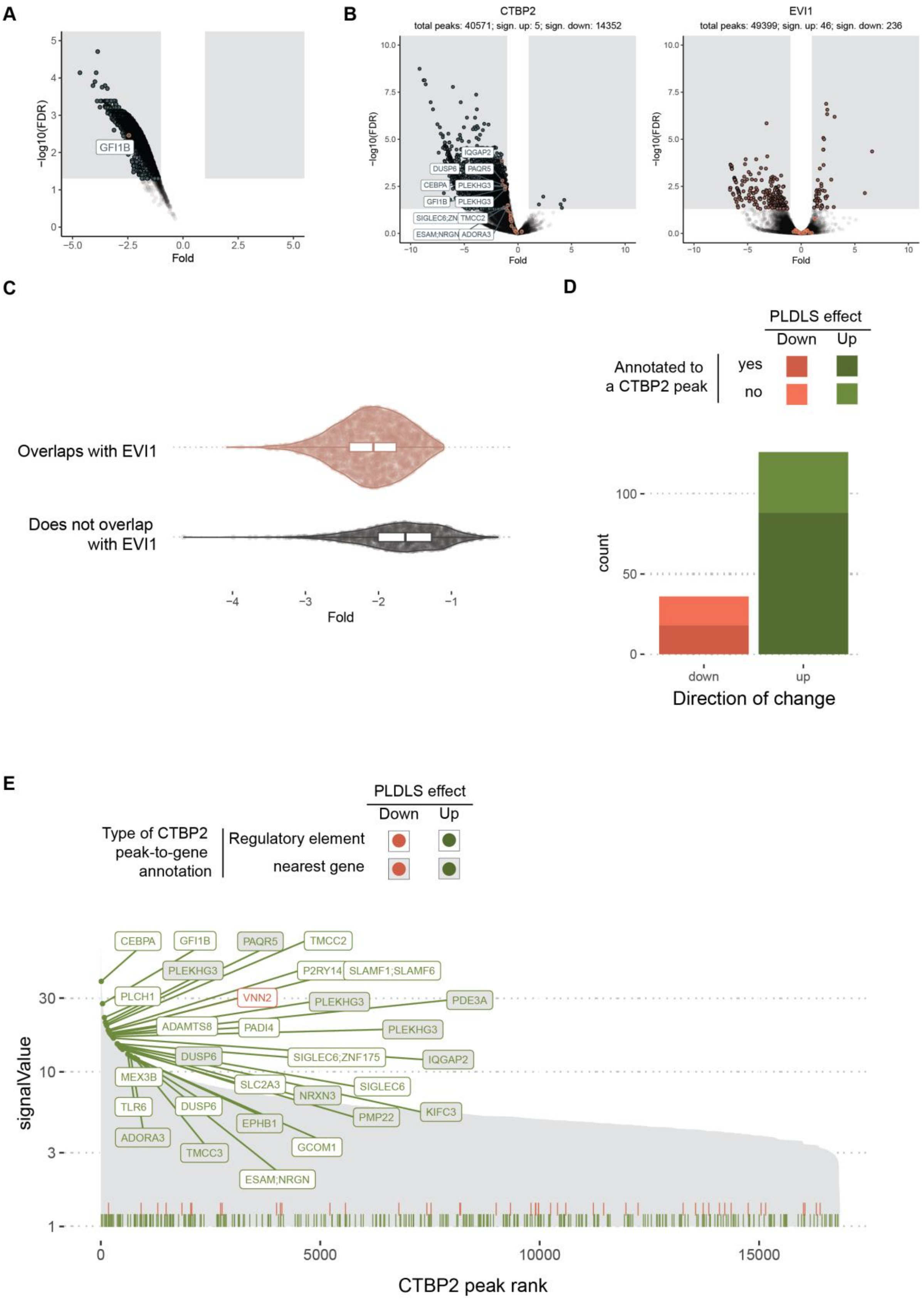
A
GFI1B
−log10(FDR)
Fold
B
CTBP2
total peaks: 40571; sign. up: 5; sign. down: 14352
IQGAP2
DUSP6
PAQR5
CEBPA
PLEKHG3
GFI1B
PLEKHG3
SIGLEC6;ZN
TMCC2
ESAM;NRGN
ADORA3
EVI1
total peaks: 49399; sign. up: 46; sign. down: 236
C
Overlaps with EVI1
Does not overlap with EVI1
Fold
D
PLDLS effect
Down
Up
Annotated to a CTBP2 peak
yes
no
count
down
up
Direction of change
E
PLDLS effect
Down
Up
Type of CTBP2 peak-to-gene annotation
Regulatory element
nearest gene
CEBPA
GFI1B
PAQR5
TMCC2
PLEKHG3
P2RY14
SLAMF1;SLAMF6
PLCH1
VNN2
PLEKHG3
PDE3A
ADAMTS8
PADI4
PLEKHG3
DUSP6
SIGLEC6;ZNF175
IQGAP2
MEX3B
SLC2A3
NRXN3
SIGLEC6
TLR6
DUSP6
PMP22
KIFC3
EPHB1
ADORA3
TMCC3
GCOM1
ESAM;NRGN
signalValue
CTBP2 peak rank

Figure S6: Supplement to Figure 3

Fig S6A. Volcano plot of differential CTBP2 binding site occupancy (by ChIP-seq) analysis (DiffBind) in 4x PLASS vs 4x PLDLS -transduced MUTZ3 cells (5281 out of 5634 peaks with FDR < 0.05; n = 2).

Fig S6B. Volcano plot of differential CTBP2 and EVI1 binding (by ChIP-seq) analysis (DiffBind) in MUTZ3 cells that were transduced with dox-inducible 4xPLDLS expression. Dox-induced versus untreated bulk cell populations s are compared at 72hrs; n = 2 per group. Regions from panel S6E are labelled in both plots. The number of peaks is indicated above the plots.

Fig S6C. Fold-change of CTBP2 peaks in response to 4x PLDLS treatment in MUTZ3. Peaks are grouped according to whether they overlap with EVI1 peaks (n = 4026), or not (n =1608).

Fig S6D. Significantly differentially expressed (DE) genes (adj. P-value <0.05 and absolute fold-change > 2) upon 4x PLDLS treatment in MUTZ3 cells (n = 2, 72 hrs post-TD, 162 genes in total). The fraction of DE genes that are bound by CTBP2 are highlighted in dark. Peak-to-gene annotation is based firstly on whether the peak directly overlaps with a putative regulatory element associated to gene expression, and if it does not overlap with such an element, to the nearest protein-coding gene.

Fig S6E. CTBP2 peaks are ranked (x-axis) based on their enrichment over input (y-axis). The top-30 peaks annotated to genes from figure C based on highest CTBP2 binding are labelled. The distribution of all PLDLS-target-gene-annotated-peaks is visualised above the axis. 342 peaks could be annotated to a upregulated PLDLS target gene (88 unique genes in total, on average 3.9 peaks per gene) and 47 to a downregulated PLDLS target gene (18 unique genes in total, or 2.6 peaks per gene on average).

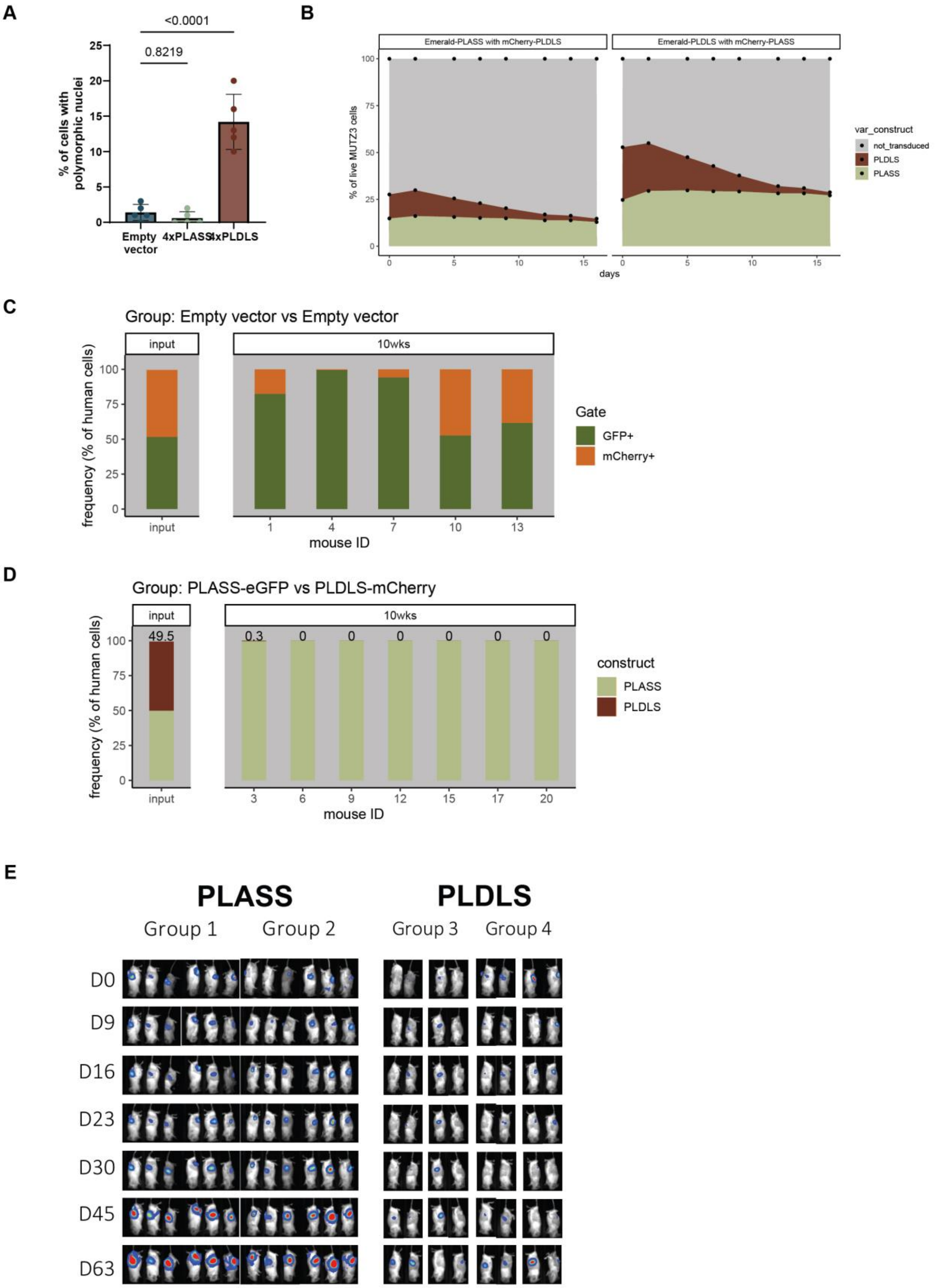
A
<0.0001
0.8219
% of cells with polymorphic nuclei
Empty vector
4xPLASS
4xPLDLS
B
Emerald-PLASS with mCherry-PLDLS
Emerald-PLDLS with mCherry-PLASS
% of live MUTZ3 cells
days
var_construct
not_transduced
PLDLS
PLASS
C
Group: Empty vector vs Empty vector
input
10wks
frequency (% of human cells)
mouse ID
Gate
GFP+
mCherry+
D
Group: PLASS-eGFP vs PLDLS-mCherry
input
10wks
49.5
0.3
frequency (% of human cells)
mouse ID
construct
PLASS
PLDLS
E
PLASS
PLDLS
Group 1
Group 2
Group 3
Group 4
D0
D9
D16
D23
D30
D45
D63

Figure S7. Supplement to Figure 4/5

Fig S7A. Counts of polymorphic (differentiated) nuclei as illustrated by arrows in panel E. Significance is tested with one-way ANOVA with multiple comparisons (Empty vector as reference; mean + SD plotted)

Fig S7B. Flow-cytometric analysis of MUTZ3 cells that were transduced with 4xPLDLS and 4X PLASS and subsequently mixed in a 50:50 ratio. Data is presented as a fraction of live cells. For Fig. 5D, the frequency of PLASS or PLDLS within the total transduced fraction is determined.

Fig S7C. Flow-cytometric analysis of input and 5 mice transplanted with SB1690 with Empty vector-Emerald and Empty vector -mCherry mixed in a 1:1 ratio.

Fig S7D. Flow-cytometric analysis of input and 7 mice transplanted with SB1690 with PLASS-Emerald and PLDLS -mCherry mixed in a 1:1 ratio.

Fig S7E. Luminescence scans of mice transplanted with MUTZ3-Luciferase +4xPLASS or MUTZ3-Luciferase +4x PLDLS vectors at indicated days after transplantation

Supplementary Table 1

- **Oligos** > all used oligos in the study
- **Chipseq_tracks** > description of all chipseq tracks used per figure panel (all available on GEO)
- **Chipseq_antibodies_protocols** > all antibodies per chip track and the used protocol per track
- **DataAvailability_PerFigure** > description of all datasets associated with this publication deposited in public repositories (GEO, ProteomeXchange, Github and ZENODO).
- **ColonyAssays_WithinGroup** >
    - Results of two-way ANOVA on within-group comparisons for Fig 4B.
    - Results of two-way ANOVA on within-group comparisons for Fig 5A.

Uncropped western blots for panel S1A

S1A

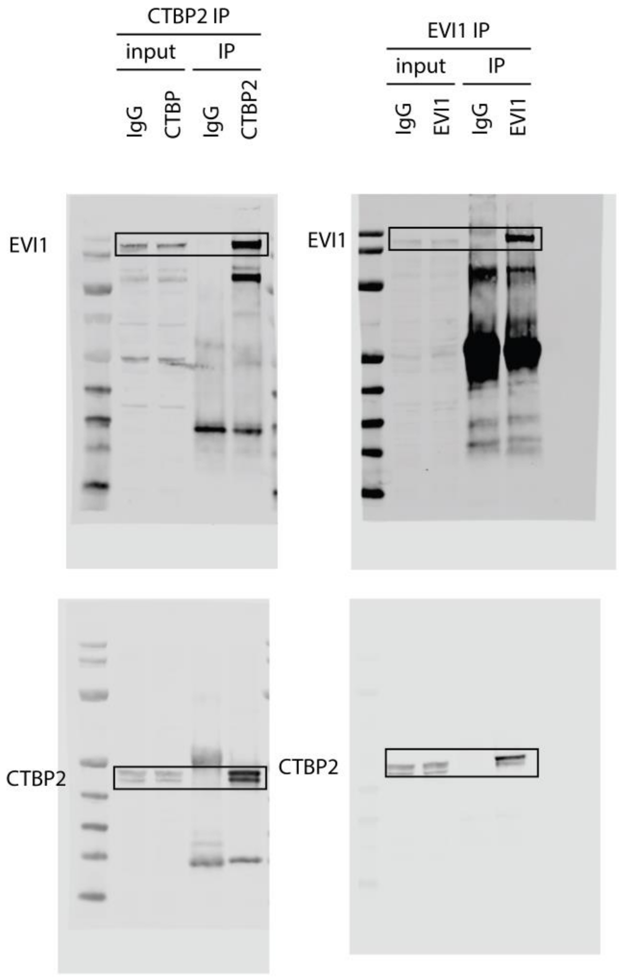

Supplement: Supplementary file 1 — Figs. S1 to S7 Legend for table S1 Uncropped Western blots for panel S1A [file sciadv.adk9076_sm.pdf]
